# Cardiac Masses and Pseudomasses: An Overview about Diagnostic Imaging and Clinical Background

**DOI:** 10.3390/medicina60010070

**Published:** 2023-12-29

**Authors:** Corrado Tagliati, Marco Fogante, Anna Palmisano, Federica Catapano, Costanza Lisi, Lorenzo Monti, Giuseppe Lanni, Federico Cerimele, Antonio Bernardini, Luca Procaccini, Giulio Argalia, Paolo Esposto Pirani, Matteo Marcucci, Alberto Rebonato, Cecilia Cerimele, Alessandra Luciano, Matteo Cesarotto, Manuel Belgrano, Lorenzo Pagnan, Alessandro Sarno, Maria Assunta Cova, Fiammetta Ventura, Luana Regnicolo, Gabriele Polonara, Lucia Uguccioni, Alessia Quaranta, Liliana Balardi, Alessandro Barbarossa, Giulia Stronati, Federico Guerra, Marcello Chiocchi, Marco Francone, Antonio Esposito, Nicolò Schicchi

**Affiliations:** 1 Radiologia, AST Pesaro Urbino, 61121 Pesaro, Italy; corradotagliati@gmail.com (C.T.); alberto.rebonato@ospedalimarchenord.it (A.R.); 2 Maternal-Child, Senological, Cardiological Radiology and Outpatient Ultrasound, Department of Radiological Sciences, University Hospital of Marche, 60126 Ancona, Italy; giulio.argalia@ospedaliriuniti.marche.it (G.A.); paolo.espostopirani@ospedaliriuniti.marche.it (P.E.P.); 3 Experimental Imaging Center, IRCCS San Raffaele Scientific Institute, Via Olgettina 60, 20132 Milan, Italy; palmisano.anna@hsr.it (A.P.); esposito.antonio@unisr.it (A.E.); 4 School of Medicine, Vita-Salute San Raffaele University, Via Olgettina 58, 20132 Milan, Italy; 5 Department of Biomedical Sciences, Humanitas University, via Rita Levi Montalcini 4, 20072 Milan, Italy; federica.catapano@hunimed.eu (F.C.); costanza.lisi@humanitas.it (C.L.); lorenzo.monti@humanitas.it (L.M.); marco.francone@hunimed.eu (M.F.); 6 IRCCS Humanitas Research Hospital, via Manzoni 56, Rozzano, 20089 Milan, Italy; 7 Radiologia, ASL 4 Teramo, 64100 Teramo, Italy; giuseppe.lanni@aslteramo.it (G.L.); fdr.cerimele@gmail.com (F.C.); antonio.bernardini@aslteramo.it (A.B.); luca.procaccini93@gmail.com (L.P.); 8 U.O.C. di Radiodiagnostica, Ospedale Generale Provinciale di Macerata, 62100 Macerata, Italy; matteomail654@gmail.com; 9 Dipartimento di Biomedicina e Prevenzione, Universiy of Roma Tor Vergata, 00133 Roma, Italy; cec.cerimele@gmail.com (C.C.); alessandraluciano.med@outlook.it (A.L.); marcello.chiocchi@gmail.com (M.C.); 10 Department of Radiology, Azienda Sanitaria Universitaria Giuliano Isontina Ospedale di Cattinara, 34149 Trieste, Italy; matteocesarotto3@gmail.com (M.C.); lorenzo.pagnan@gmail.com (L.P.); alessandro.sarno88@gmail.com (A.S.); 11 Department of Medical, Surgical and Health Sciences, University of Trieste, 34151 Trieste, Italy; mbelgrano@units.it (M.B.); m.cova@fmc.units.it (M.A.C.); 12 Department of Radiology, IRCCS-INRCA Hospital, 60125 Ancona, Italy; fiam.ventura@gmail.com; 13 Department of Neuroradiology, University Hospital of Marche, 60126 Ancona, Italy; luana.regnicolo@ospedaliriuniti.marche.it; 14 Department of Specialized Clinical Sciences and Odontostomatology, Polytechnic University of Marche, 60126 Ancona, Italy; g.polonara@staff.univpm.it; 15 Emodinamica e Cardiologia Interventistica, AST Pesaro Urbino, 61121 Pesaro, Italy; lucia.uguccioni@ospedalimarchenord.it; 16 Cardiologia, Distretto Sanitario di Civitanova Marche, AST 3, 62012 Civitanova Marche, Italy; alessiaquaranta84@gmail.com; 17 Health Professions Area, Diagnostic Technical Area, University Hospital of Marche, 60126 Ancona, Italy; liliana.balardi@ospedaliriuniti.marche.it; 18 Cardiology and Arrhythmology Clinic, Department of Cardiological Sciences, University Hospital of Marche, 60126 Ancona, Italy; alessandro.barbarossa@ospedaliriuniti.marche.it (A.B.); giulia.emily.stronati@gmail.com (G.S.); federico.guerra@ospedaliriuniti.marche.it (F.G.); 19 Cardiovascular Radiological Diagnostics, Department of Radiological Sciences, University Hospital of Marche, 60126 Ancona, Italy; nicolo.schicchi@ospedaliriuniti.marche.it

**Keywords:** cardiac masses, pseudomasses, non-neoplastic lesions, benign tumors, malignant neoplasms

## Abstract

A cardiac lesion detected at ultrasonography might turn out to be a normal structure, a benign tumor or rarely a malignancy, and lesion characterization is very important to appropriately manage the lesion itself. The exact relationship of the mass with coronary arteries and the knowledge of possible concomitant coronary artery disease are necessary preoperative information. Moreover, the increasingly performed coronary CT angiography to evaluate non-invasively coronary artery disease leads to a rising number of incidental findings. Therefore, CT and MRI are frequently performed imaging modalities when echocardiography is deemed insufficient to evaluate a lesion. A brief comprehensive overview about diagnostic radiological imaging and the clinical background of cardiac masses and pseudomasses is reported.

## 1. Introduction

Cardiac lesions can be subdivided into pseudomasses and true masses. The latter can be categorized into non-neoplastic masses and neoplastic ones, which can be classified into benign and malignant, as reported in the 2021 WHO classification [1]. These lesions can be recognized as intracavitary, myocardial, valvular, pericardial lesions or as paracardiac masses involving atrioventricular groove or pericardiophrenic angles. 

Cardiac ultrasonography is the first-line imaging modality, but when the nature of the lesion is not clearly understood or the precise localization and the relationship with surrounding tissues and coronary arteries need to be evaluated, computed tomography (CT) and magnetic resonance (MR) imaging are performed [2]. Moreover, the increasing use of coronary CT angiography to evaluate coronary artery disease results in more frequent observation of incidental findings [3]. PET/CT could be useful when a lymphoma or a malignant lesion is suspected, and sometimes it could determine false positive findings as it is the case of lipomatous hypertrophy of the inter-atrial septum in which there is metabolically active brown adipose tissue [4]. 

Many cardiac masses consist of pseudomasses, non-neoplastic lesions and benign tumors, and lesion characterization is very important to manage the patients appropriately. Large lesions with ill-defined borders, tissue heterogeneity, nodular pericardial involvement and multiplicity are suspicious findings for malignancy.

## 2. Pseudomasses

### 2.1. Normal Intracardiac Structures

Normal cardiac structures or embryologic remnants can sometimes raise suspicion of tumor or thrombus with echocardiography, especially if prominent, but are generally easily characterized with MR imaging or CT given the large field of view and high spatial resolution (Table 1). 

**Figure 1:**
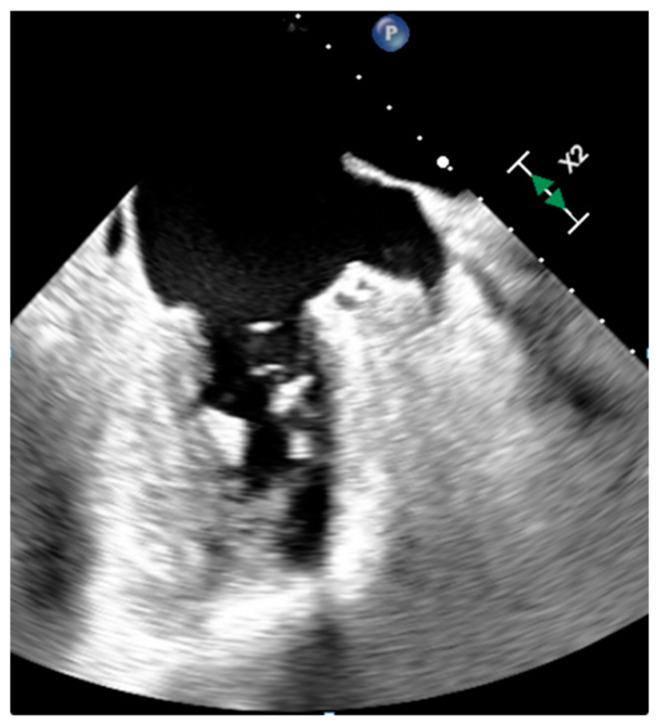
Warfarin ridge at transesophageal echocardiography.

Coumadin ridge or warfarin ridge or left atrial ridge is a normal elongated structure with a bulbous tip in the left atrium that lies between the left atrial appendage and left upper pulmonary vein (Figure 1). A prominent ridge can be mistaken for a thrombus or tumor, but it usually has an echogenicity that is similar to surrounding cardiac structures and shows mobility with underlying tissue. CT attenuation, MR signal intensity and contrast enhancement are like those of the normal surrounding atrial wall. Sometimes the coumadin ridge may be a substrate for paroxysmal atrial fibrillation. Rarely, tumors such as fibroelastoma and myxoma may originate from this ridge [5,6,7].

**Figure 2:**
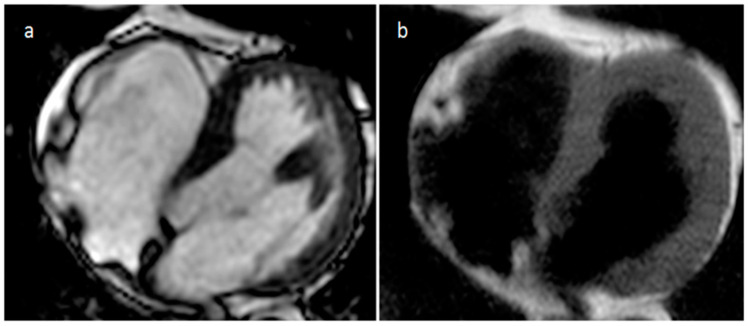
Axial cine MR sequences (**a**) and T1-weighted images (**b**) in a patient with prominent crista terminalis.

Crista terminalis or terminal crest is a smooth and well-defined structure in the posterolateral right atrial wall near the superior vena cava and it can measure about 3 to 6 mm. It is present in every heart, and it is frequently not visualized during echocardiography. However, a prominent crista terminalis may be misinterpreted as a cardiac mass or pseudomass. It is represented by a linear echogenic ridge on echocardiograms, and at CT and MR it shows the same attenuation or signal intensity and contrast enhancement of the surrounding atrial wall (Figure 2). It is sometimes associated with right atrial tachyarrhythmias (cristal tachycardias) [8].

The taenia sagittalis or sagittal bundle is the most prominent pectinate muscle in the right atrium (RA). It shows a single trunk in about 60% of the population, multiple trunks in about 20%, and it is absent in about 20% of cases. The taenia sagittalis is usually not visible during cardiac ultrasonography. During CT and MR the taenia sagittalis is visualized as a linear ridge that goes from the crista terminalis to the right atrial appendage (RAA). During CT and MR, it shows similar attenuation or signal intensity and enhancement to those of the surrounding right atrial wall. It may be a substrate for atrial flutter or fibrillation [9].

**Figure 3:**
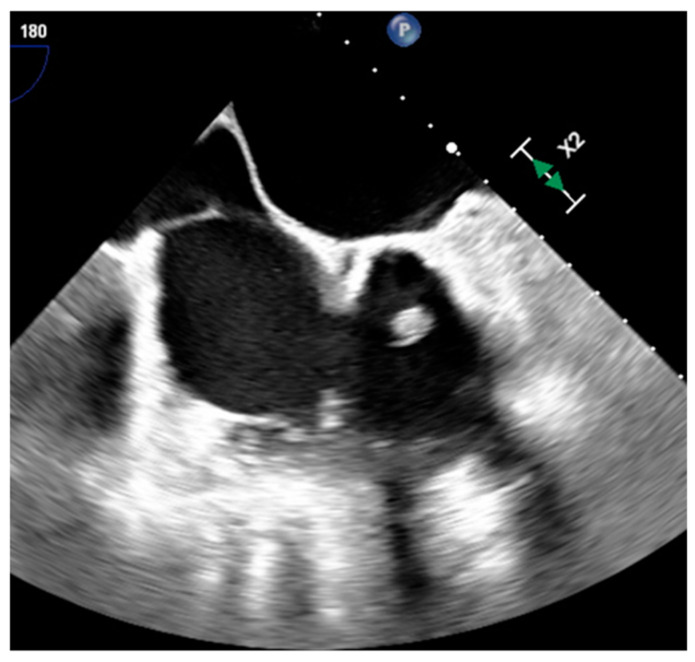
A prominent eustachian valve at transesophageal echocardiography.

The Chiari network, Chiari remnant or right atrial net is a net-like structure in the RA over the IVC ostium but can also be represented by a linear band between the eustachian and/or thebesian valves and the crista terminalis, or a prominent eustachian valve (Figure 3). The Chiari network can be seen in about 10% of the population. When visualized during echocardiography, it is highly mobile and echogenic. It can favor supraventricular arrhythmia and it is associated with patent foramen ovale and paradoxical embolism. However, the Chiari network could reduce the risk of embolization from deep leg vein thrombosis [10].

**Figure 4:**
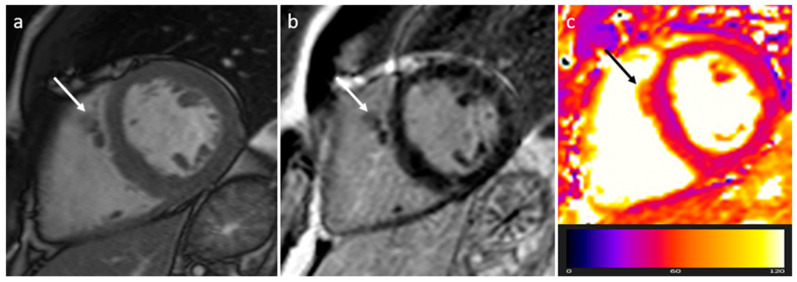
Right ventricle pronounced muscolar ridge which extends from the basal ventricular septum to the free wall on cine images (**a**), late gadolinium enhancement MR sequence (**b**), and T2 mapping image (**c**).

The moderator band is the most pronounced muscular ridge in the RV, and it extends from the ventricular septum to the free wall at the base of the anterior papillary muscle. It carries the right bundle branch of the AV bundle, and when conspicuous it can be mistaken for a thrombus at echocardiography. However, during CT and MR the moderator band shows attenuation, signal intensity, and contrast enhancement similar to those of the RV (Figure 4). Infrequently a hypertrophied or atypically located papillary muscle can be confused with a mass during echocardiography. However, during MR or CT the entire course and attachment of the muscle can be simply evaluated, therefore allowing for the correct diagnosis [11,12].

### 2.2. Lipomatous Hypertrophy of the Inter-Atrial Septum (LHIS or LHAS)

**Figure 5:**
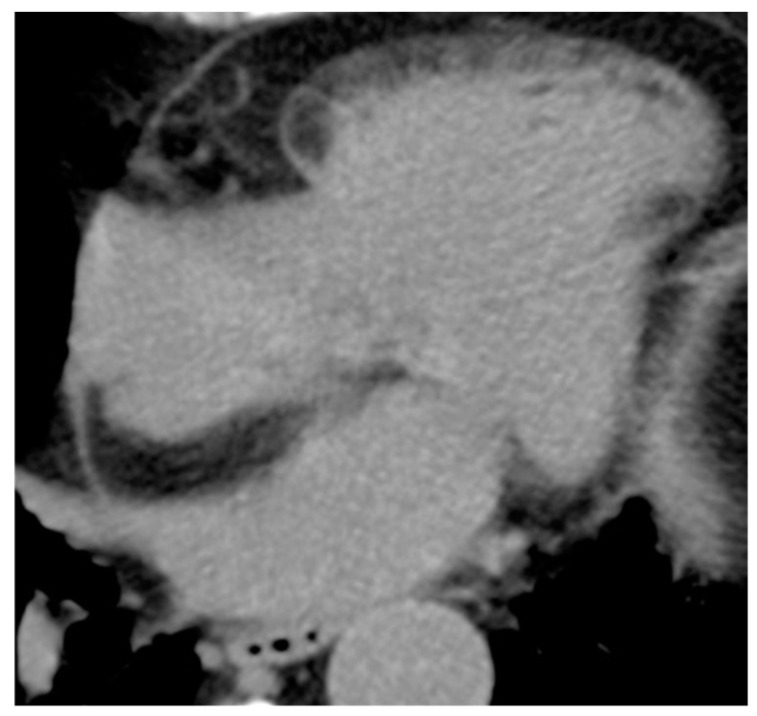
81-year-old female patient with lipomatous hypertrophy of the interatrial septum in CT scan.

**Figure 6:**
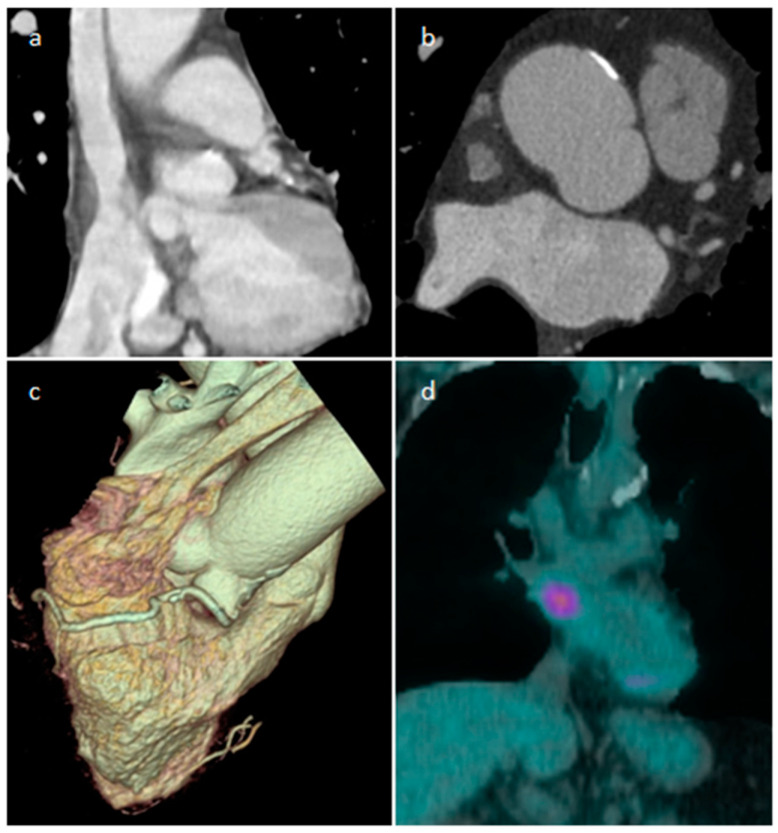
A 65-year-old male patient with lipomatous hypertrophy of the interatrial septum on coronal and axial CT images (**a**,**b**), volume rendering (**c**), which showed focal uptake of FDG in PET/CT scan (**d**).

LHIS is a benign unencapsulated hyperplasia of lipocytes resembling brown fat in the fold of the septum secundum, sparing the fossa ovalis which forms part of the septum primum, producing the typical barbell or dumbbell appearance (Figure 5). Interatrial Septum (IS) thickness > 2 cm suggests the diagnosis of LHIS. Extensive LHIS can also involve the posterior wall of the right atrium and crista terminalis. Cardiac lipoma is encapsulated, affects the fossa ovalis and represents the main differential diagnosis. LHIS metabolically active brown adipose tissue can show 18F-FDG uptake during Positron Emission Tomography (Figure 6). LHIS is usually asymptomatic. However, sometimes it can cause atrial arrhythmias or, rarely, superior vena cava compression. Surgery is indicated if there are signs of superior vena cava syndrome, or if there are severe intractable arrhythmias [13,14,15]. 

## 3. Non-Neoplastic Masses

### 3.1. Thrombus

**Figure 7:**
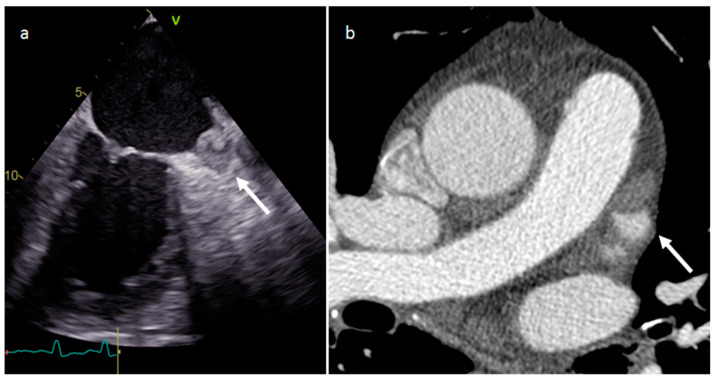
Left atrium appendage thrombus at transesophageal echocardiography (**a**) and CT (**b**).

**Figure 8:**
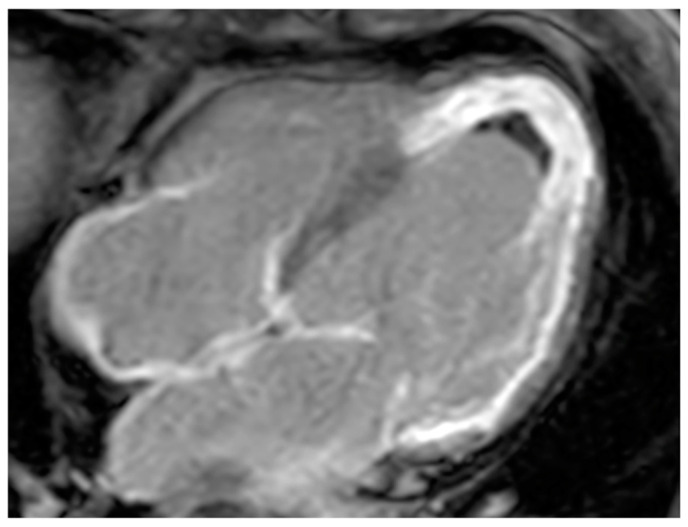
A 67-year-old female patient with left ventricle thrombus related to myocardial infarction on late gadolinium enhancement MR sequence.

Thrombus is the most common cardiac mass. It is usually identified in the left atrium in patients with atrial fibrillation, atrial dilatation, mitral stenosis or previous mitral valve surgery (Table 2) (Figure 7). After myocardial infarction thrombus can be found adjacent to a left ventricle hypokinetic region (Figure 8). Occasionally, thrombi can be located in the RA or in vena cava, especially in patients with enlarged right chambers and with central venous lines. They are usually broad-based and move synchronously with the adjacent wall during the cardiac cycle. During CT a thrombus shows low attenuation on delayed phase acquisition, and it shows absence of iodine uptake on dual-energy CT iodine map. Cardiac MR is the most accurate imaging method in differentiating cardiac thrombi and tumors. T1- and T2-weighted signal characteristics vary depending on the age of a thrombus. Usually, thrombus does not show contrast enhancement. Late gadolinium enhancement (LGE) imaging can sometimes show a hypointense border and a brighter central zone. However, organized chronic thrombus may rarely enhance peripherally due to its fibrous content. Frequently patients are asymptomatic, and thrombus identification is necessary in order to start anticoagulation treatment and prevent possible embolic events [16,17,18].

### 3.2. Vegetations

**Figure 9:**
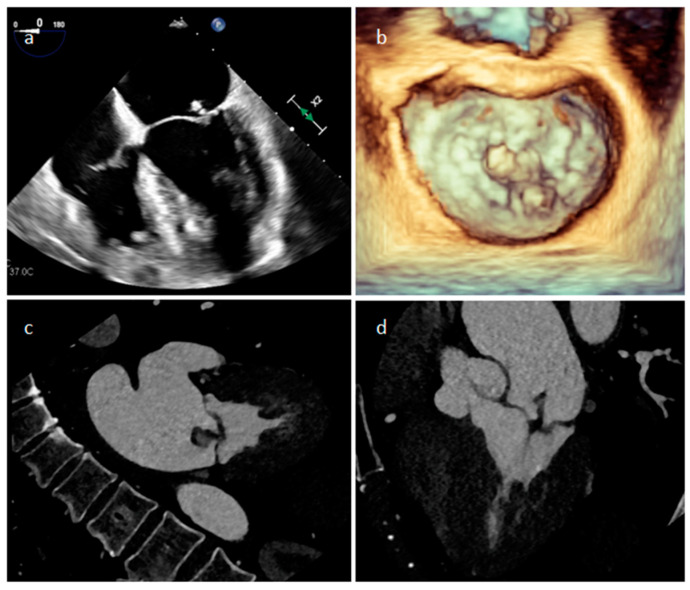
A mitral valve vegetation at 2D (**a**) and 3D (**b**) transesophageal echocardiography, and at CT (**c**,**d**).

**Figure 10:**
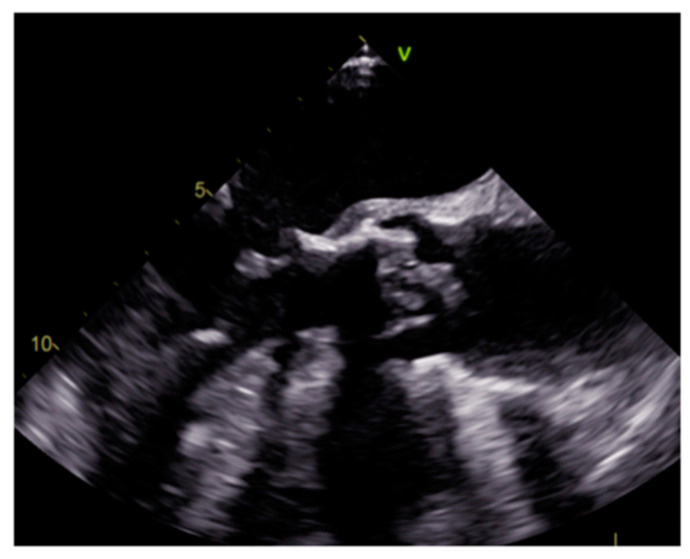
Prosthetic aortic valve infective endocarditis with vegetations at transesophageal echocardiography.

**Figure 11:**
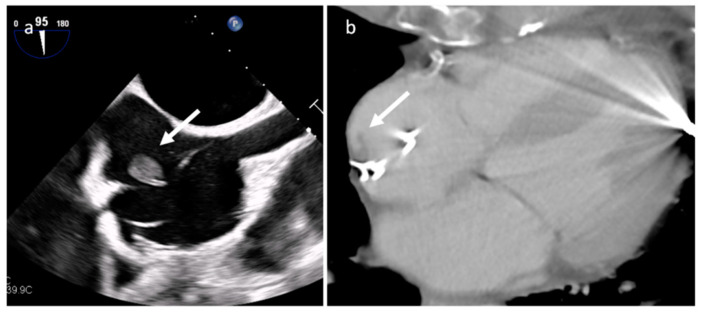
Electro-catheter endocarditis at transesophageal echocardiography (**a**) and CT (**b**).

Endocarditis is usually due to an infectious etiology and is characterized by leaflet destruction and often valvular incompetence (Figure 9). Either a native or prosthetic cardiac valve may be affected (Figure 10). Moreover, electrocatheters can be involved (Figure 11). The modified Duke criteria are used for diagnosis. Echocardiography is the first-line imaging modality for patients with suspected vegetations. Vegetations are highly mobile lesions, and usually do not enhance. However, peripheral rim enhancement on LGE imaging rarely can be observed. Moreover, adjacent myocardial enhancement could denote perivalvular abscess, irreversible myocardial damage, or fibrosis. CT and MR are very useful to evaluate complications. In fact, severe cases may be characterized by perivalvular extension with paravalvular abscesses, mycotic pseudoaneurysm or fistula formation. Moreover, total-body CT should be performed to evaluate distant septic embolization. Proper diagnosis is vital because prompt management with antibiotic administration and surgery is crucial. Sometimes small verrucous vegetations can be found in sterile endocarditis secondary to inflammation (Libman-Sacks endocarditis), such as in patients with systemic lupus erythematosus [19].

### 3.3. Mitral Annular Calcification and Its Caseous Degeneration 

**Figure 12:**
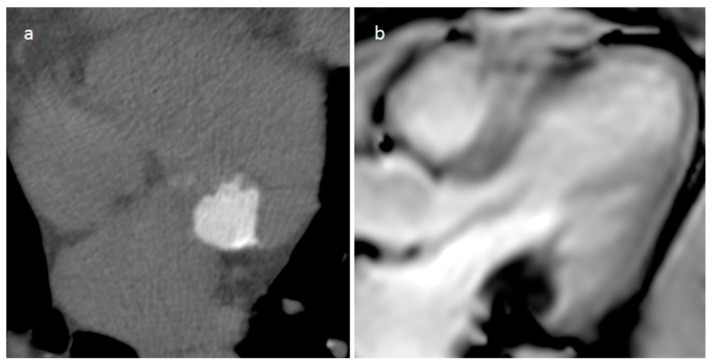
A 74-year-old female patient with mitral annular calcification on precontrast-phase CT image (**a**) and cine sequence (**b**).

Mitral annular calcification is an immobile mass, generally located in the inferolateral portion of the mitral anulus, caused by annular fibers calcium deposits, and rarely it can affect the whole annulus and press the myocardium (Figure 12). Imaging modalities show typical calcium deposition, and the fibrotic rim in which the calcified core is enclosed can show a peripheral LGE imaging hyperenhancement [20]. Rarely, this lesion can evolve into caseous degeneration (or liquefaction necrosis), and in this case the lesion core contains a mixture of cholesterol, fatty acids, and amorphous eosinophilic infiltrate with a surrounding envelope that enfolds lymphocytes, macrophages, and multiple necrotic areas with calcifications [21]. It is usually asymptomatic. However, symptoms can be caused by related complications such as mitral stenosis or regurgitation, infective endocarditis, and embolization. The central fluid proteinaceous and fatty content can show high signals on both T1- and T2-weighted sequences. Calcifications around and within the lesion are hypointense on MRI imaging but are better evaluated with CT. Peripheral LGE imaging hyperenhancement consistent with a fibrous cap is observed. 

### 3.4. Pericardial Cyst

**Figure 13:**
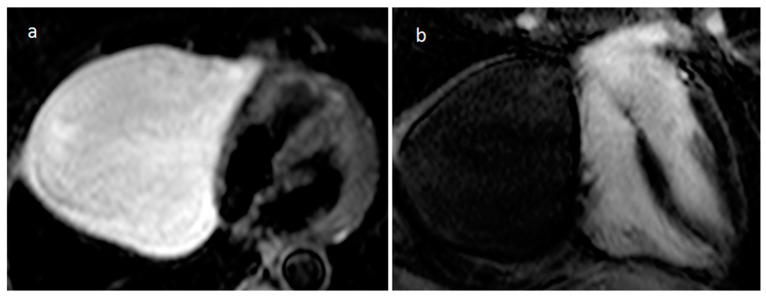
A 70-year-old female patient with a large right pericardiophrenic angle pericardial cyst on axial T2 STIR (**a**) and four-chamber view late gadolinium enhancement (**b**) MR imaging.

A pericardial cyst is usually congenital in etiology, but sometimes it is acquired following pericarditis. It is often placed in the right pericardiophrenic angle (70%), and less frequently in the left one (25%) (Figure 13). Sometimes intracystic septations or enhancing walls can be detected. It is usually asymptomatic, and imaging is useful to evaluate lesion stability. However, rarely larger cysts can determine right cardiac chambers compression, and patients may present with congestive heart failure symptoms or retrosternal pain. In these cases, surgery could be indicated [22]. 

### 3.5. Coronary Artery Aneurism (CAA)

**Figure 14:**
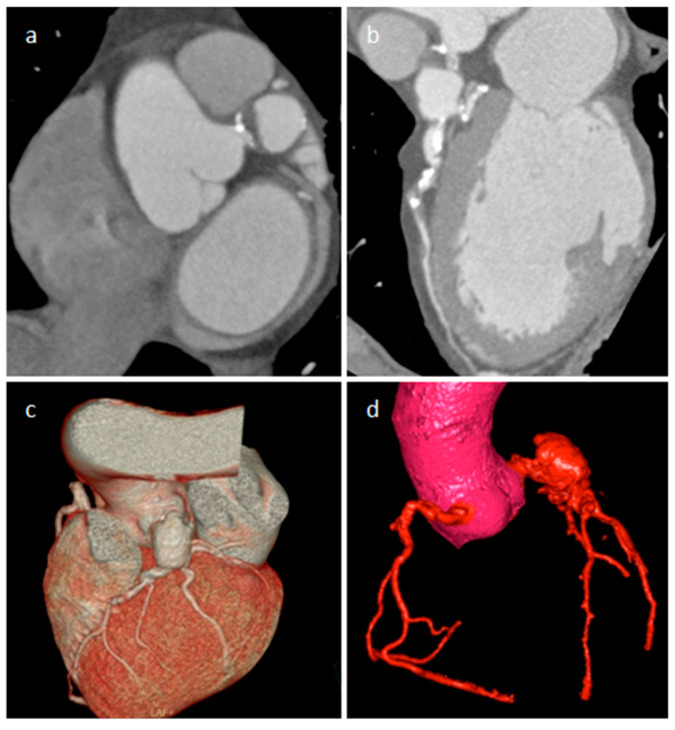
A 65-year-old male patient with a partially thrombosed giant aneurysm of the left main coronary artery on short-axis view (**a**), multiplanar reconstruction (**b**) and volume rendering (**c**,**d**) CT images.

CAA may be mistaken for mass at cardiac ultrasonography, particularly if there is extensive peripheral thrombus. A saphenous vein coronary artery bypass grafting aneurysm can be detected 10–15 years after surgery, and it can be completely thrombosed. CT or MRI allows confident diagnosis as well as delineation of the size and extent of the aneurysm (Figure 14). Usually asymptomatic, rarely it can cause myocardial ischemia or infarction, fistula formation or can evolve into rupture. A coronary artery aneurysmal dilatation can also be found in the setting of a long-standing coronary artery fistula, as a reduced vascular resistance and increased flow caused by an abnormal communication of the coronary artery with a pulmonary artery, cardiac chamber, coronary sinus, or cardiac vein determine a dilatation of the involved artery. The right coronary artery is more frequently involved, and a left-right shunt occurs in most lesions. Coronary CT angiography is useful to evaluate the size of the dilatation, the presence of a concomitant thrombus, and the fistula anatomy [23].

### 3.6. Cardiac Chambers Aneurysm and Pseudoaneurysm

**Figure 15:**
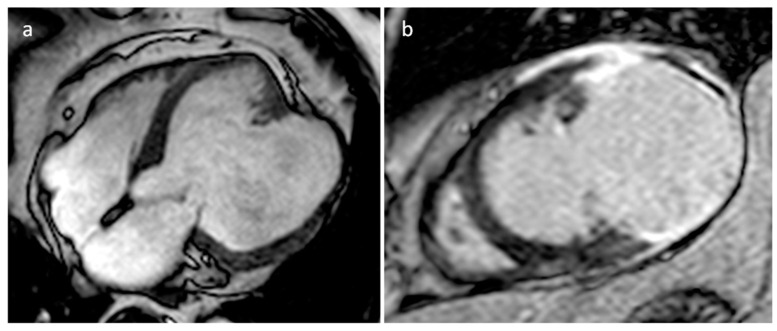
A 67-year-old male patient with huge left ventricle aneurysm after myocardial infarction on four-chamber cine sequence (**a**) and axial late gadolinium enhancement MR sequence (**b**).

Aneurysm and pseudoaneurysms of the ventricles or atria may occur as complications of cardiac surgery, or secondary to infarction, trauma, or infection (more frequently endocarditis), and they could be occasionally mistaken for a focal mass [23]. CT and MRI are very useful to define the origin of these blood-filled structures (Figure 15). 

### 3.7. IgG4-Related Disease (IgG4-RD)

**Figure 16:**
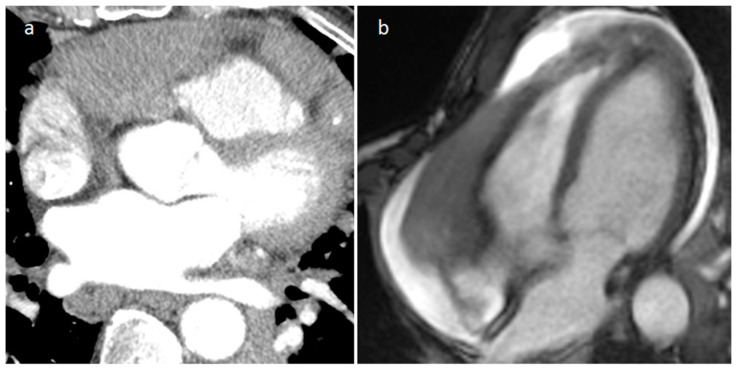
Right atrioventricular groove IgG4-related disease on arterial CT phase (**a**) and on cine MR sequence (**b**).

IgG4-RD is a fibro-inflammatory disease that can involve nearly any organ and tissue. It is a systemic condition characterized by IgG4-positive lymphoplasmacytic tissue that infiltrates with subsequent fibrotic aftereffects. Common manifestations include major salivary and lacrimal gland enlargement, autoimmune pancreatitis, and retroperitoneal fibrosis [24]. Cardiovascular involvement includes coronary stenosis or dilatation surrounded by a tumor-like lesion or periarterial thickening, pericardial thickening and effusion, constrictive pericarditis, cardiac masses (Figure 16), valvular stenosis and regurgitation, periaortitis and aortic aneurysm, pulmonary artery stenosis and pulmonary hypertension, and carotid arteries stenosis. FDG PET/CT can evaluate the activity and severity of the disease and can identify the best biopsy site. Glucocorticoids are considered the first-line agents and azathioprine is frequently used as additional therapy. Moreover, pericardial drainage, pericardiectomy, valve replacement and surgical resection of masses could be performed [25]. 

### 3.8. Sarcoidosis

**Figure 17:**
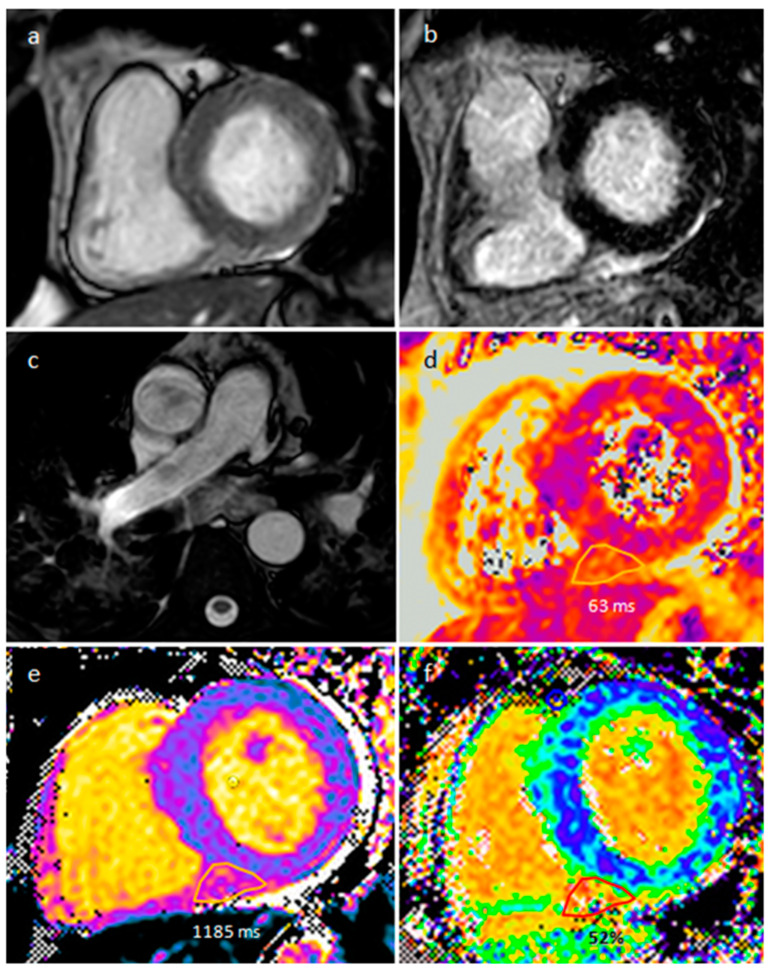
A 57-year-old male patient with sarcoidosis on axial cine sequence (**a**), short tau inversion recovery sequence (**b**), hilar and pulmonary involvement on balanced-steady-state free precession images (**c**), T2 mapping (**d**), native T1 mapping (**e**) and post-contrast T1 mapping (**f**) sequences (normal T2 value < 50 ms and normal native T1 value < 1045 in our site).

Sarcoidosis is a multisystem chronic granulomatous disease, and cardiac involvement seems to be responsible for about 10% of sarcoidosis-related deaths. The basal septum is mainly involved, and several reports showed cardiac tumor-like lesions in the ventricular septum, right ventricle, left and right atria [26]. MR imaging can reveal myocardial delayed enhancement, and myocardial oedema can be found in active sarcoidosis (Figure 17). In young patients with heart failure, new atrioventricular (AV) block, and cardiac arrest cardiac sarcoidosis should be excluded. In fact, cardiac MR imaging should be performed as an early screening tool for myocardial involvement in patients presenting with AV block requiring immediate pacing for bradycardia, and FDG PET/CT segmental uptake is the main cardiac sarcoidosis feature related to active disease. Moreover, early initiation of glucocorticoids in patients with second degree AV block could avoid pacemaker implantation [27].

### 3.9. Foreign Body

Foreign bodies can manifest as cardiac masses. They can be either iatrogenic (gossypiboma, catheter, inferior vena cava filters, guide wires, pacemaker leads) or traumatic (chopstick, bullets). Diagnosis is usually incidental in an asymptomatic patient who previously performed a surgical or intravascular procedure. However, sometimes patients can be highly symptomatic with chest pain and dyspnea caused by heart failure, perforation or obstruction of vessels or cardiac chambers [28,29,30,31]. The foreign body is encapsulated in fibroinflammatory tissue, clotted blood and/or thrombus, and the shape and type of the object is sometimes difficult to recognize by imaging. Treatment strategies can vary depending on the patient’s symptoms and the foreign body location and nature. However, surgical removal is often considered in these patients, especially when the patient is symptomatic or there are risks of perforation, embolization, or infection.

### 3.10. Hematoma

**Figure 18:**
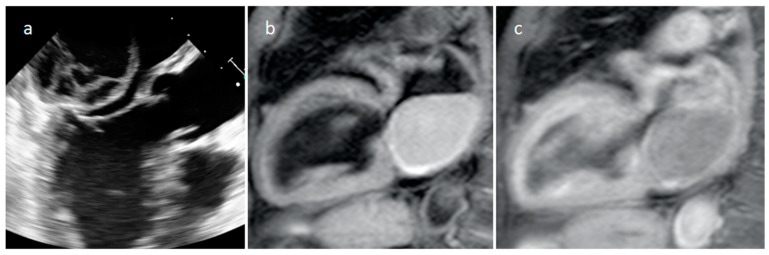
Echocardiography (**a**), T1-weighted (**b**) and T1-weighted gadolinium-enhanced MR sequences (**c**) in a patient with spontaneous acute left atrial hematoma.

Hematoma is usually detected in patients with previous surgery, and some clips can be found around it. Pericardial hematoma is classified as acute in the first week after surgery or trauma, subacute between one and four weeks, and chronic if the hematoma persistent for more than a month. Hematoma can appear mass-like on echocardiography and as mixed density on CT (Figure 18). Density is usually high in the acute phase and then it slowly decreases. On MR it may have different signals, depending on its age and sequence used. It does not show internal enhancement, and chronic hematoma can show internal calcifications. However, sometimes it can show rim-enhancement. A hematoma usually resolves without sequela, however unfrequently it can persist and sometimes become larger over time as blood breakdown products can cause localized inflammatory reactions, which might be at the base of additional bleeding and further inflammation, causing a persisting cycle that evolve into the so called chronic expanding hematomas. On MRI, a chronic expanding hematoma can show a hypointense peripheral capsule and heterogeneous central contents indicating the presence of fresh and old blood. After heart injuries hematomas can cause symptoms within a few days with a potential risk of hemodynamic compromise. In fact, fast or slow-growing space-occupying lesions can cause hemodynamic instability due to compression of great vessels and cardiac chambers. However, patients with post-surgical pericardial hematoma are usually asymptomatic, and serial follow-up examinations are useful to evaluate disease progression, stability or regression [32,33,34].

### 3.11. Echinococcus Cyst 

**Figure 19:**
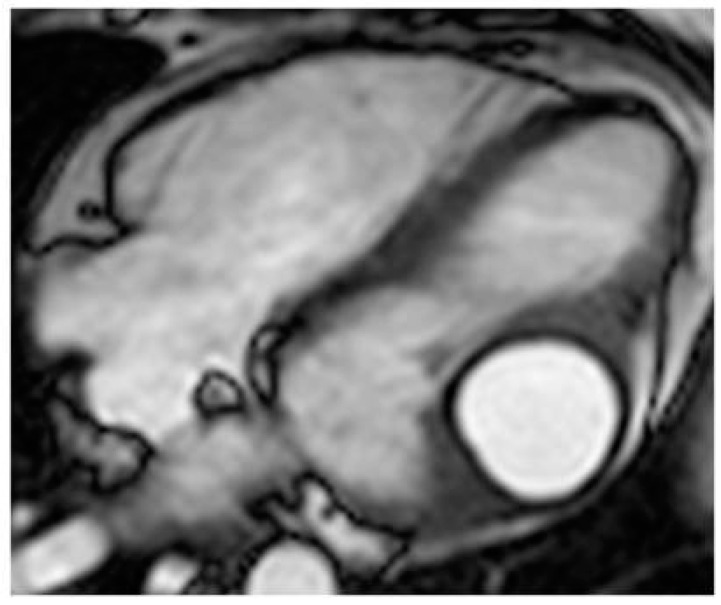
A 26-year-old female patient with left ventricular myocardium echinococcus cyst, which is hyperintense with a dark rim on cine MR sequence.

The most frequent locations of echinococcosis are the liver and lungs, followed by muscles, bone and kidneys. Cardiac hydatid occurs in about 1% of cases. Usually, the cyst is identified in the LV free wall or RV free wall. Rarely, the pericardium, IVS or atria can be involved [35]. Frequently, cardiac lesions are associated with the liver and/or lung [36]. Diagnosis can frequently be made by echocardiography recognizing a cyst with multiple septa and daughter cysts in a patient with positive laboratory tests. However, CT and MR are useful to define a possible involvement of surrounding structures and for presurgical planning (Figure 19). Cardiac cysts can be asymptomatic or can be associated with dyspnea, chest pain, ventricular arrhythmias, valvular dysfunction, and outflow tract obstruction [37]. Cardiac echinococcosis is usually managed by combining the use of antiparasitic drugs with surgical cyst excision. 

## 4. Benign Tumors

### 4.1. Myxoma

**Figure 20:**
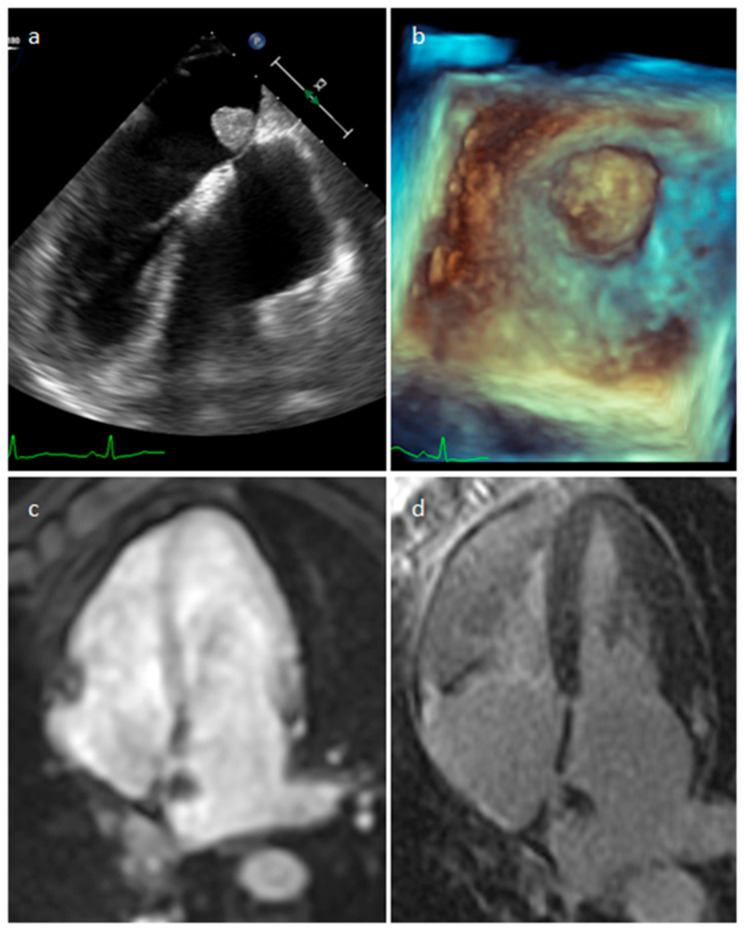
Left atrial myxoma at 2D (**a**) and 3D (**b**) transesophageal echocardiography, perfusion (**c**) and late gadolinium enhancement (**d**) MR imaging.

**Figure 21:**
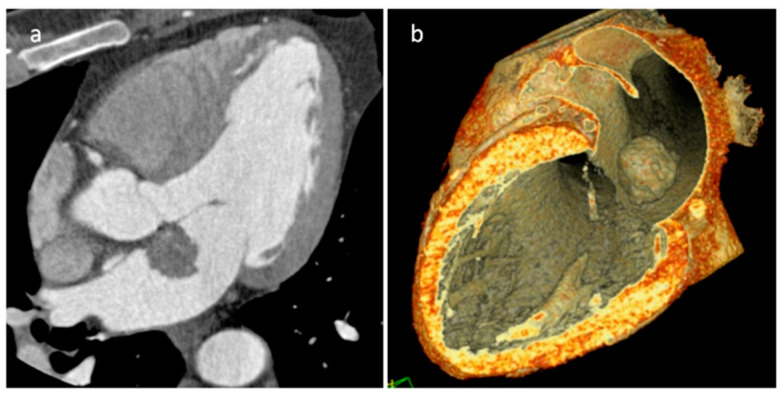
Left atrial myxoma on three-chambers view (**a**) and three-dimensional volume rendering (**b**) CT images in a 45-year-old male patient.

**Figure 22:**
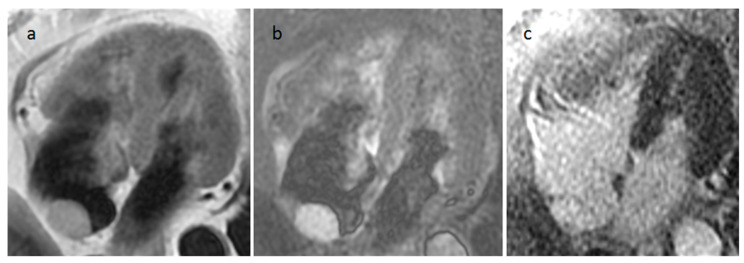
Right atrial myxoma isointense on T1-weighted images (**a**), hyperintense on T2-weighted images (**b**), hyperintense and mildly heterogeneous on late gadolinium enhancement sequence (**c**).

Myxomas are the most frequent primary cardiac tumors (35%) and are most located in the left atrium (70%) (Table 3) (Figure 20, Figure 21 and Figure 22). A minority of them (10%) are associated with Carney complex (cardiac and skin myxomas, lentiginosis, and endocrine tumors) [38]. Typical echocardiographic characteristics are represented by polypoid or papillary mobile atrial masses attached to the IAS (Table 3). LGE images show heterogeneous enhancement due to possible cystic degeneration, necrosis, hemorrhage, calcification, and associated thrombus. Native T1 and T2 relaxation times and ECV values are higher with respect to normal myocardium, owing to greater fluid content and interstitial space [39]. Polypoid myxoma determines more frequently intracardiac obstruction, and papillary-type myxomas are more frequently associated with embolization. Surgical excision is necessary and needs to be performed quickly after the diagnosis in order to reduce the risk of embolization. Recurrences can be observed in about 15% of patients with Carney complex and in about 5% of other patients, therefore close follow-up is recommended [40,41]. 

### 4.2. Papillary Fibroelastoma (PFE)

**Figure 23:**
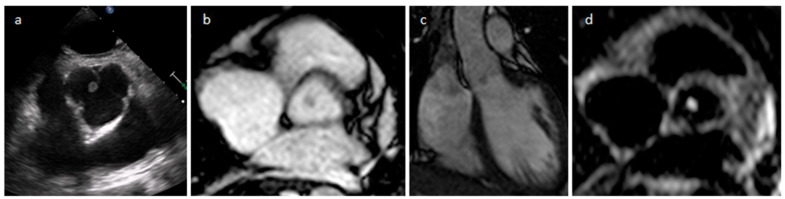
A 28-year-old male patient with an aortic cusp fibroelastoma during transesophageal echocardiography (**a**), iso/hypointense on axial (**b**) and left ventricular outflow tract (**c**) cine sequences, and hyperintense on axial T2-weighted images (**d**).

**Figure 24:**
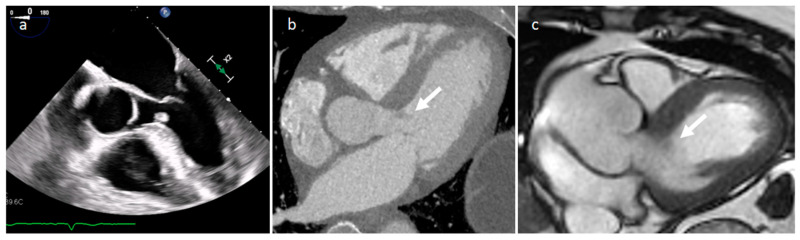
A left ventricular outflow tract fibroelastoma of about one centimeter during transesophageal echocardiography (**a**), CT (**b**) and cine MR imaging (**c**). These images show that a fibroelastoma can be more easily evaluable during ultrasonography.

PFE is the most frequent cardiac valve tumor. It is usually smaller than 1.5 cm and mostly located in the vascular surface of the aortic valve, or less frequently in the atrial surface of the mitral valve (Figure 23 and Figure 24). When the mass is too small to be detected the only abnormality that may be seen is the adjacent turbulent flow on cine images. It is usually asymptomatic, however embolic events have been reported from attached thrombi or fragmentation. Surgical excision is recommended for lesions > 1 cm, left sided tumors, or in patients with cerebral embolic events or angina from coronary ostial obstruction [42]. 

### 4.3. Lipoma

**Figure 25:**
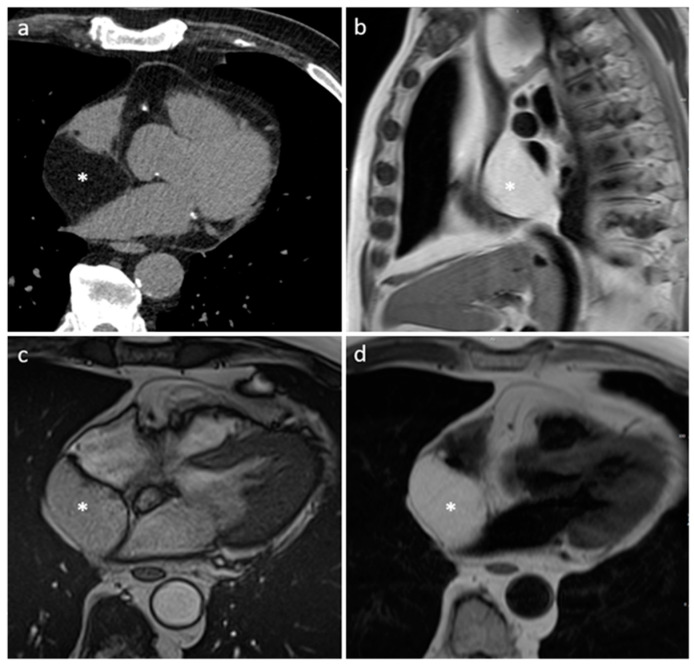
Right atrium (*) posterior wall capsulated ovoid fatty mass in CT scan (**a**), T1-weighted images (**b**,**d**) and cine (**c**) MR images.

**Figure 26:**
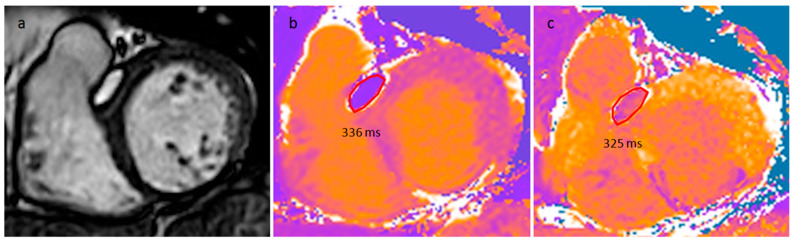
A 52-year-old woman with an interventricular septum lipoma on cine sequences (**a**), native T1 mapping (**b**) and post-contrast T1 mapping (**c**) sequences.

Lipomas are made up of mature adipose tissue and most commonly arise in the subepicardium. On echocardiography they tend to be well circumscribed, homogenous, and hyperechoic [43]. On cine MR imaging lipomas typically show “india ink” artifact, also known as black boundary artifact or type 2 chemical shift artifact (Figure 25 and Figure 26). Usually, they are asymptomatic and do not need treatment. However, they can rarely determine coronary arteries compression when subepicardial, arrhythmias when originating from the mid-myocardial layer or outflow obstruction when placed in the subendocardium [44].

### 4.4. Rhabdomyoma

**Figure 27:**
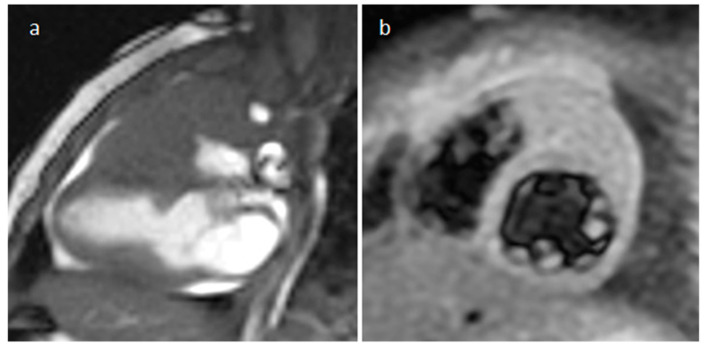
A neonate with a basal anterior and mid-cavity anterior left ventricular wall mass on balanced steady-state free precession sequence (**a**) and short tau inversion recovery MR sequence (**b**).

Rhabdomyomas are the most frequent pediatric primary cardiac tumors, and they are usually identified in utero or in children under 1 year of age. About 70% of them are associated with tuberous sclerosis in which rhabdomyomas are multiple in roughly 75% of cases. Multiple cardiac masses recognized at prenatal echocardiography suggests tuberous sclerosis. They are usually intramyocardial or intracavitary and frequently involve the left ventricular wall (Figure 27). Most lesions spontaneously regress in early childhood; therefore, surgery is needed only in case of inflow or outflow obstruction or refractory arrhythmias [45].

### 4.5. Fibroma 

Cardiac fibromas are the second most common pediatric tumor and are frequently diagnosed in the first year of life. A minority of cardiac fibromas are associated with Gorlin syndrome represented by basal cell carcinomas, brain tumors and skeletal abnormalities. They are usually identified as single tumors in the ventricular wall that do not regress; therefore, surgery is necessary. Iso-hypointensity on T1-weighted MR images, hypointensity with possible hyperintense periferic rim on T2-weighted MR images, hypointensity on first-pass perfusion, hyperintensity with possible hypointense rim on LGE imaging in association with clinical data, particularly the age of presentation, frequently allow mass characterization. However, the information about coronary anatomy and tumor blood supply provided by CT are very important for surgical planning [46,47].

### 4.6. Hemangioma

Hemangiomas are benign tumors composed of endothelial cells lining venous blood vessels, more frequently of the cavernous type. They are typically solitary and show evident gradual, nodular, peripheral to central post-contrast enhancement. They usually show reassuring features, as well-defined contours without invasion of contiguous structures. They are usually asymptomatic, but sometimes can cause arrhythmias, dyspnea, chest pain, congestive heart failure, or recurrent pericardial effusion especially for pericardial lesions. Very rarely they were associated with the so called Kasabach–Merritt syndrome characterized by recurring thrombocytopenia and consumptive coagulopathy. They can arise anywhere in the heart, but with a predilection for the ventricle walls, RA, and pericardium. Tumor surgical resection is the treatment of choice, and LA hemangiomas may be resected like cardiac myxomas. Also, pericardial lesions can be simply surgically removed. However, for myocardial hemangioma the risk of complications is higher. If a feeding vessel is identified, coil embolization could be considered, but it is obviously very dangerous for possible induced ischemic events [48].

### 4.7. Lymphangioma

Lymphangiomas are very rare primary cardiac tumors, characterized by multicystic/multilocular lesions. They are usually hypointense in T1-weighted MR imaging, however few case reports showed high T1 signal intensity due to proteinaceous fluid content. They can sometimes show cystic walls and septa post-contrast enhancement. Lymphangiomas are frequently large lesions with a mean size of 6 cm at diagnosis, and therefore they can cause symptoms, such as arrhythmia, dyspnea, chest pain and heart failure. Surgical excision is usually performed [49,50,51]. 

### 4.8. Erdheim-Chester Disease (ECD)

**Figure 28:**
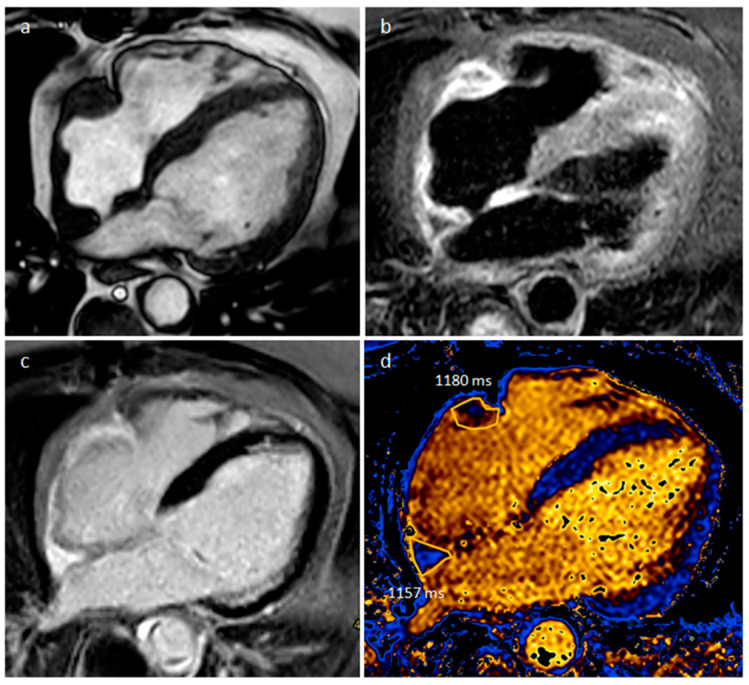
A 56-year-old male patient with right atrial and right atrioventricular groove masses on four-chamber cine sequence (**a**), short tau inversion recovery (**b**), double-inversion recovery (**c**) and native T1 mapping (**d**) MR sequences (normal native T1 value < 1045 in our site).

ECD is a rare histiocytosis that was recently recognized as a hematopoietic neoplastic disorder [52]. Diagnosis of ECD is made with histology and phenotype of histiocytes in appropriate clinical and radiological context [53]. Symmetric diaphyseal and metaphyseal osteosclerosis in the legs is nearly always present. Cardiovascular involvement is frequently underdiagnosed and occurs in at least 50% of patients [54]. The most frequent cardiovascular manifestations of ECD are infiltration of the AV groove with enhancing soft tissue often encasing the coronary arteries, pericardial thickening, enhancement and effusion, nodular or mass-like thickening of the right atrial wall and periaortic sheathing (Figure 28). One-third of patients will develop retroperitoneal fibrosis, especially around the kidneys and ureters. Central nervous system involvement, diabetes insipidus and/or exophthalmos with retro-orbital mass occur in 20% to 30% of patients. The most frequent cutaneous manifestation is xanthelasma, generally involving the eyelids or periorbital spaces. Most patients with ECD need systemic treatment at diagnosis, usually with BRAF- and/or MEK-inhibitors. However, sometimes the disease can be simply monitored, particularly in asymptomatic patients with nonvital single-organ involvement or in case of minimally symptomatic disease [55].

### 4.9. Solitary Fibrous Tumor (SFT)

SFT is a rare spindle cell mesenchymal lesion that usually has a pleural origin. However, primary intrapericardial SFT are reported, and vimentin, CD34, CD99, and STAT6 immunostaining positivity can allow its diagnosis. SFT is usually benign and coronary arteries are not infiltrated at coronary CT angiography. However, about 15% of SFTs are locally aggressive or malignant. Complete resection is the recommended treatment, and long-term follow-up is necessary, as local recurrences are described [56].

### 4.10. Teratoma

Teratomas are the most common germ cell tumors and are usually benign. They are generally diagnosed in childhood or fetal life as multiloculated intrapericardial lesions with cystic and solid components typically placed near the root of the pulmonary artery or aorta. Usually asymptomatic, they can sometimes cause RA or superior vena cava compression. Focal contrast enhancing areas or signs of adjacent structures infiltration are suggestive for malignant teratomas. Complete surgical excision is recommended. Recurrences are possible, more frequently when the resection is incomplete or the teratoma is immature. Therefore, long-term follow-up is necessary with periodic imaging evaluations and alfa-fetoprotein levels monitoring [57,58].

### 4.11. Schwannoma

Schwannomas are rare, usually benign tumors, which likely arise from the cardiac branch of the vagus nerve or the cardiac plexus, and therefore in many cases they involve the right heart. However, primary malignant schwannomas have also been rarely reported. Moreover, malignant psammomatous melanotic schwannomas (MPMS) represent a little portion of all schwannomas that can be are difficult to distinguish from melanoma because of histologic and radiologic similarities, but immunohistochemical differences can be demonstrated. Typical imaging characteristics consist of non-infiltrating well-circumscribed lesions, frequently containing cystic components, sometimes with hemorrhage, and usually without calcifications. Sometimes they show a peculiar target appearance on T2-weighted images due to a central fibrocollagenous portion enclosed by a peripheral myxomatous component. The post-contrast internal enhancement is usually slow: small lesions more frequently show intense homogeneous enhancement, and larger lesions usually enhance heterogeneously. Schwannomas are often asymptomatic and can be incidentally detected on chest X-ray, chest computed tomography or coronary CT angiography. However, patients can experience nonspecific symptoms like fatigue, dyspnea, cough and/or facial edema due to large lesions that may determine great vessels, mediastinal structures or heart chambers compression. Benign schwannomas are surgically resected in order to obtain a confirmatory histopathological diagnosis, without needing any adjuvant therapy [59,60,61].

### 4.12. Paraganglioma

**Figure 29:**
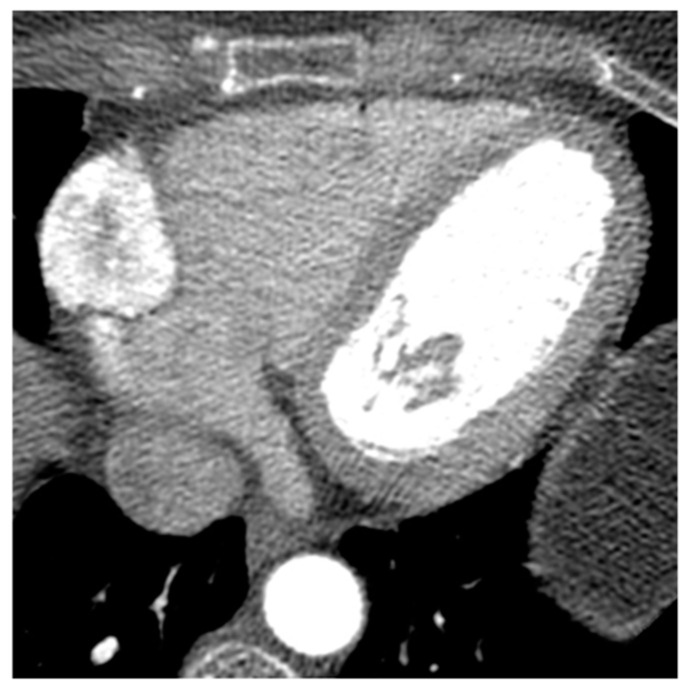
A right atrioventricular groove paraganglioma with predominant peripheral enhancement due to central necrosis on arterial phase CT image.

Paragangliomas are neuroendocrine tumors deriving from neural crest cells localized outside adrenal glands. A large minority of paragangliomas occur in the chest, and most of them are detected in the posterior mediastinum. Therefore, cardiac paragangliomas are extremely rare. Urine and blood tests in order to detect catecholamines and catecholamines metabolites are necessary to make the diagnosis (Figure 29). MR shows a hypo-isointense signal on T1 weighted images and an intensely high signal on T2, the so called “light bulb bright signal”, with “salt and pepper” appearance on both T1 and T2 weighted sequences; salt is determined by hemoglobin degradation products due to intralesional hemorrhage and pepper is caused by flow voids related to high vascularity. Paragangliomas show immediate and intense contrast enhancement after contrast media injection, similar to arterial vessels. They frequently show peripheral LGE which depends on tumor necrosis. However, homogeneous enhancement can be shown by smaller lesions. Patients can report palpitations, flushing, headaches and may also be hypertensive, as up to 50% of paragangliomas secrete catecholamines. Paragangliomas are highly vascularized, and biopsy is contraindicated in order to avoid life-threatening bleeding. Pathology is the gold standard for paraganglioma diagnosis; however, histopathology differentiates benign from malignant lesions with difficulty, and the detection of distant metastasis is the only reliable sign of malignancy. Frequently, curative resection of paragangliomas is difficult to obtain due to complicated connections with the adjacent coronary arteries. Recurrence rates are reported of up to 25%. Therefore, long-term follow-up of the patient is required with regular CT or MR scans. Moreover, a proper family screening is required, as about 30% of paragangliomas are familial [62].

## 5. Malignant Tumors

### 5.1. Secondary Tumors-Metastasis

**Figure 30:**
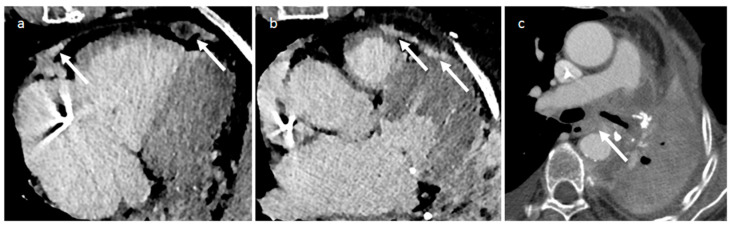
CT images of a 66-year-old female patient with multiple pericardial metastases (white arrows—images (**a**,**b**)) and lymphadenopathies (white arrow—image (**c**)) who previously underwent left lobectomy for lung adenocarcinoma.

**Figure 31:**
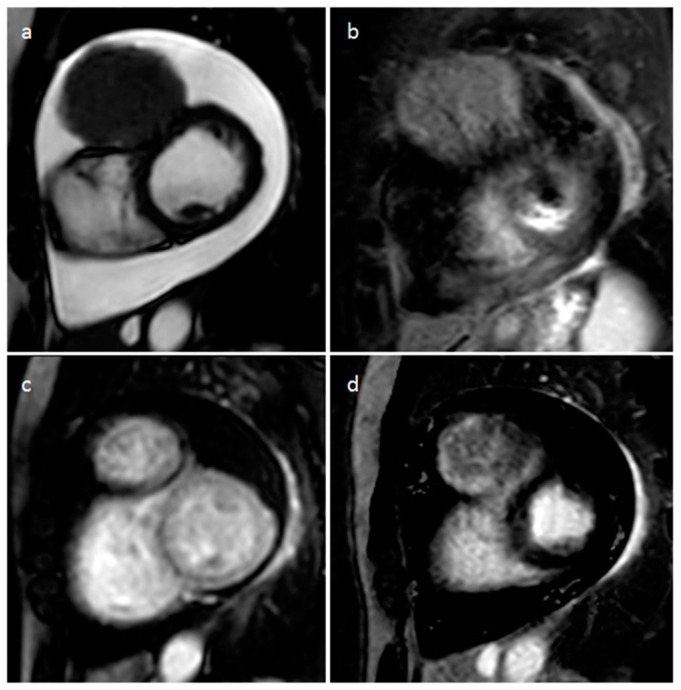
Uterine sarcoma visceral pericardial metastasis on two-chamber steady-state free precession (**a**), short tau inversion recovery (**b**), first-pass perfusion (**c**) and late gadolinium enhancement MR sequences (**d**).

**Figure 32:**
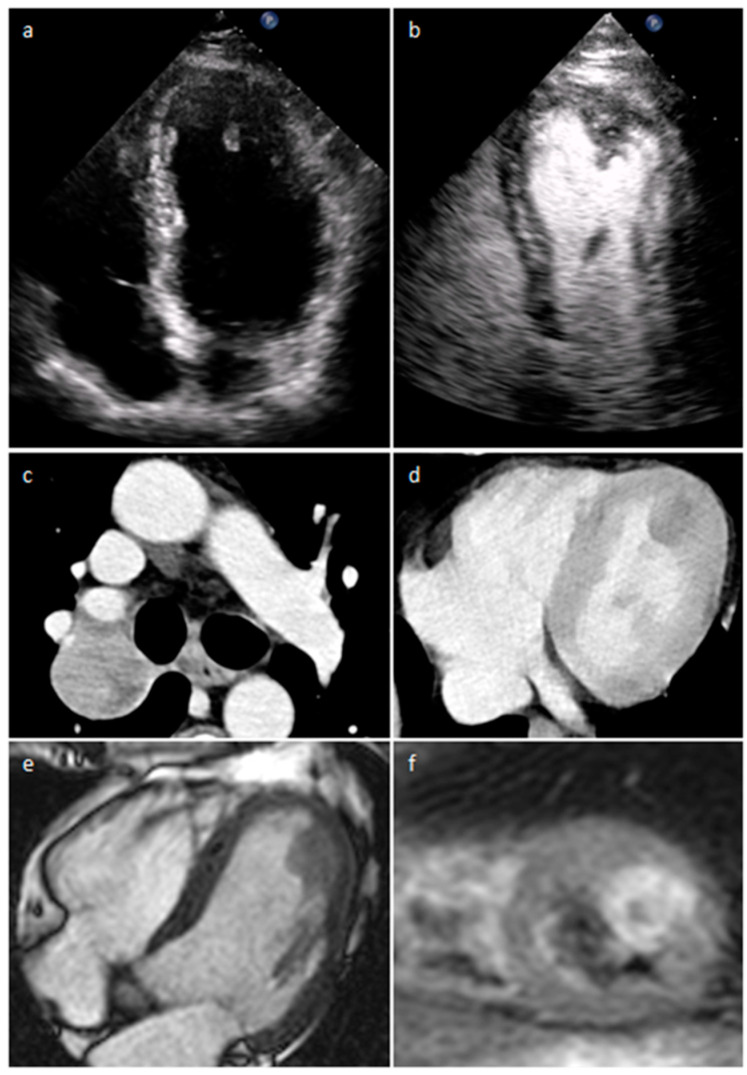
Left ventricle metastasis on precontrast (**a**) and postcontrast (**b**) transthoracic echocardiogram images; primitive lung lesion (**c**) and cardiac metastasis on venous phase CT (**d**); cardiac secondary tumor on four-chamber cine (**e**) and short-axis T2 STIR (**f**) MR imaging.

**Figure 33:**
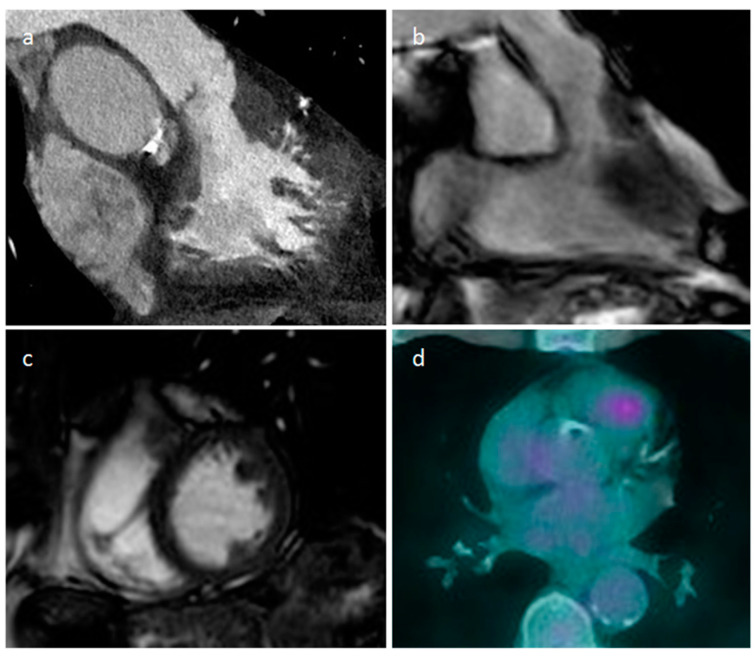
A 72-year-old male patient with right ventricular outflow tract colorectal cancer metastasis in CT scan (**a**), MR imaging (**b**,**c**) and FDG PET/CT scan (**d**).

**Figure 34:**
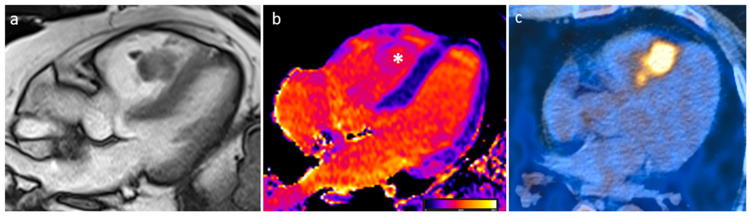
Right ventricular melanoma metastasis (*) with intermediate-high signal intensity on cine sequence (**a**), high native T1 mapping values (1600 ms) (**b**), and avid FDG uptake in PET-CT scan (**c**).

Cardiac metastasis are the most common tumors that involve the heart (Table 4). They are more frequently located in the pericardium (about 60%), epicardium (about 20%) and myocardium (about 20%) (Figure 30, Figure 31, Figure 32, Figure 33 and Figure 34). The more common primary tumors are breast and lung cancers that spread by direct extension or retrograde lymphatic diffusion thereby causing pericardial lesions and effusion. Melanoma typically involves the myocardium by hematogenous spread. Transvenous extension with inferior vena cava neoplastic thrombosis and intracavitary right atrium mass growth can occur in hepatocellular and renal cell carcinomas. Cardiac metastases generally show low signal intensity on MR T1-weighted images, high signal intensity on T2-weighted images and heterogeneous contrast enhancement. However, melanoma metastasis and hemorrhagic lesions can show high signal intensity on T1 weighted sequence due to the presence of paramagnetic melanin or blood degradation products. Obviously, appropriate disease staging needs to be performed first to assess a potential oncologic treatment of cardiac metastases, and PET-CT is the recommended imaging modality in case of secondary cardiac lymphomas [63,64].

### 5.2. Primary Cardiac Sarcomas

**Figure 35:**
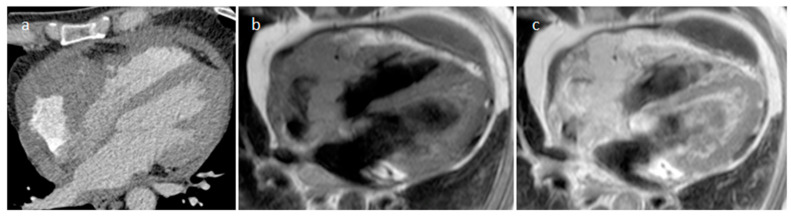
A 37-year-old male patient with a right atrial angiosarcoma at CT (**a**), on precontrast (**b**) and postcontrast (**c**) T1-weighted images.

**Figure 36:**
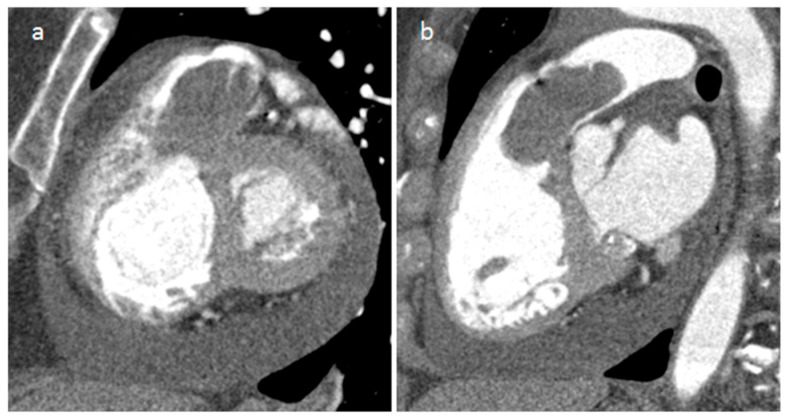
Right ventricular outflow tract and pulmonary primary cardiac leiomyosarcoma at CT (**a**,**b**).

Sarcomas are the most frequent malignant primary cardiac tumors, and they can be linked to Li–Fraumeni syndrome. The most common histotypes are angiosarcoma, undifferentiated pleomorphic sarcoma, leiomyosarcoma and rhabdomyosarcoma (Figure 35 and Figure 36). The latter frequently affects childhood, and the others usually are detected in middle adulthood. Rhabdomyosarcoma usually involves the right chambers, angiosarcoma right atrium, undifferentiated pleomorphic sarcoma and leiomyosarcoma left atrium. Sarcomas are irregular lesions with low attenuation at CT, and heterogeneous enhancement at MR perfusion imaging. Angiosarcoma tends to show stronger enhancement and more hyperenhancement on T2 weighted images compared to other sarcomas. Sarcomas are usually heterogeneously isointense on T1, heterogeneously iso-hyperintense on T2-weighted images and frequently show heterogeneous LGE. When a patient has acceptable performance status and no or limited metastatic disease, resection and/or debulking should be undertaken. Moreover, chemotherapy and/or radiation therapy is usually offered [1,65,66]. 

### 5.3. Primary Cardiac Lymphoma

**Figure 37:**
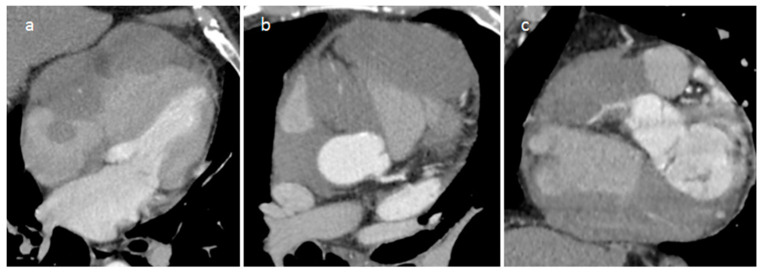
A 63-year-old male patient with right heart large B-cell lymphoma on four-chamber (**a**), axial (**b**) and short-axis CT (**c**) views.

Primary cardiac lymphomas are extranodal lymphomas that primarily involve the pericardium and/or the heart, usually the right-sided cardiac chambers, and frequently cause pericardial thickening and effusion. These lesions are thought to arise within the epicardial lymphatic network and are usually diffuse large B-cell lymphomas. Lymphomas are frequently infiltrative masses that extend along the epicardial surfaces of the heart and encase adjacent coronary arteries and/or aortic roots (Figure 37). The diagnosis can be confirmed by cytology of pericardial effusion and/or tumor tissue biopsy. MR imaging findings are the same in primary and secondary cardiac lymphomas. The most common finding in echocardiography and CT images is pericardial effusion. MR can more easily identify pericardial or right chambers masses, which usually display homogeneous iso-hypointensity on T1 weighted images, iso-hyperintensity on T2 weighted images, and none or minimum contrast enhancement. Lymphomas are densely cellular tumors which usually show evident diffusion restriction. Moreover, they are typically hypermetabolic on PET imaging. Primary cardiac lymphomas can show a good response to the monoclonal anti-CD20 antibody [67,68]. 

### 5.4. Primary Pericardial Mesothelioma

**Figure 38:**
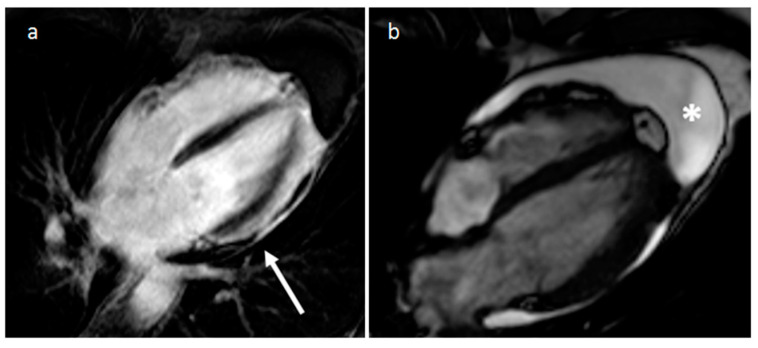
Parietal pericardium thickening (white arrow) and enhancement on late gadoliunium enhancement sequence (**a**) and cine MR images (**b**), with associated pericardial effusion (*).

Pericardial mesothelioma is a malignant proliferation of mesothelial cells, and it is the most frequent primary malignancy of the pericardium. Obviously, a direct extension of pleural mesothelioma needs to be excluded. Interestingly, asbestos exposure is described in only 15% of cases. Conspicuous pericardial thickening is usually found; however, cytologic examination is often insufficient to make a diagnosis (Figure 38). Surgical resection can be performed, and newer-generation chemotherapeutics can be administrated to the patients, but they are very rarely curative [69].

## 6. Discussion

Cardiac pseudomasses are frequently encountered in daily practice, as well as thrombus which is more prevalent in patients with atrial fibrillation or ventricular dysfunction, whereas vegetations are not so hardly found in very sick patients, such as intensive care units ones [12,19]. Cardiac tumors are rarer, and myxoma is usually considered the most common benign neoplasm. However, nowadays papillary fibroelastoma is increasingly detected thanks to high-resolution imaging which allows the diagnosis of this small valvular lesion [1]. Metastases are frequent in terminal cancer patients, and malignancies are the minority of primary cardiac neoplasms [1]. 

Transthoracic echocardiography is usually the imaging modality that detects a cardiac lesion which needs further assessment, and transesophageal examination can be useful to confirm the presence of a mass or to diagnose a specific pseudomass [70]. In fact, advanced echocardiography, such as three-dimensional, transesophageal and contrast-enhanced ultrasound can adequately evaluate size, attachment to adjacent structures and hemodynamic impact of a lesion. 

However, CT or MR are frequently performed. MR is renowned to be the best imaging modality due to the spontaneous contrast between tissue components; therefore, it is the ideal tool in such a pathology. However, MR examinations are quite long, need patients’ collaboration and some devices do not allow for performing them. Moreover, very small lesions are not so very well evaluated with MR imaging, particularly if smaller than 5 mm and mobile [71].

CT is a quick and highly available imaging technique, and it can simply assess calcifications within a lesion and coronary tree. CT could be sufficient in order to diagnose a pseudomass, a thrombus, a pericardial cyst, a vegetation, a coronary artery aneurism, a cardiac chamber aneurysm or pseudoaneurysm, a mitral annular calcification, an echinococcus cyst, or to suspect the presence of a myxoma, a papillary fibroelastoma or a lipoma. In fact, new generation CT scanners allow cardiac imaging specialists to evaluate the coronary arteries, heart, and pericardium with lower radiation doses than in the past. Moreover, in newborns, infants, and young children the high temporal resolution of new CT scanners allows a low-dose, fast and highly defined imaging acquisition. Usually, a coronary-CT angiography with retrospective acquisition and a chest venous phase acquisition are acquired. Sometimes, a delayed acquisition can be performed to evaluate the possible presence of contrast enhancement [72,73,74,75,76,77,78,79,80,81,82,83,84]. 

It is known that tissue characterization is an indisputable prerogative of MR imaging. In fact, it yields its best when a mass is intramural or not only intracavitary, allowing one to suspect that a lesion could be a fibroma, a rhabdomyoma, a paraganglioma, a hemangioma or a lymphangioma. Moreover, an infiltrative growth pattern can be suggested by CT images and confirmed at MR imaging. Usually performed MR imaging sequences are cine images, T1 and T2 black blood images, first-pass perfusion sequences, early gadolinium enhancement, postcontrast T1-weighted images, and late gadolinium enhancement. Moreover, native T1, T2 and postcontrast T1 mapping sequences can be acquired in order to calculate T1 time, T2 time and extracellular volume, with the aim of displaying them voxel by voxel on parametric maps and helping in tissue characterization [85,86,87,88,89,90].

Sometimes, PET/CT can be performed preoperatively, particularly in patients with suspected malignant lesions, in which the amount of Standardized Uptake Value (SUV) can raise or reduce the suspicion that a mass is malignant. Obviously, PET/CT can show focal extracardiac FDG uptake suspicious for metastasis or a very high SUV in case of lymphoma. However, it is more usually performed during the follow-up of malignant lesions [91,92].

When a cardiac lesion is identified, past medical history, age, gender and laboratory tests are necessary information to know, and nowadays a multimodality imaging approach is frequently important to try to reach a diagnosis. Sometimes biopsy is required, particularly in the suspicion of primary cardiac malignant lesions [93]. 

Primary malignant tumors apart from lymphoma are completely surgically resected when possible, and surgical debulking can be performed in order to improve symptoms. Myxoma needs complete resection in order to avoid embolization [94]. Papillary fibroelastomas are surgically resected when larger than 1 cm, if left sided, or in patients with cerebral embolic events or angina from coronary ostial obstruction [93]. Paraganglioma, schwannoma, teratoma, solitary fibrous tumor and vegetations are usually surgically resected [57,60,62]. The other lesions could sometimes be surgically resected when symptomatic and systemic drugs cannot treat them. Normal intracardiac structures do not require any treatment [94]. 

## 7. Conclusions

CT is very useful to confirm or exclude the presence of cardiac lesions after suspicious ultrasound findings, and it allows coronary evaluation for pre-surgical planning. Nowadays MRI is the best imaging modality for lesion characterization, and it is very useful to perform imaging follow-up of cardiac tumors. Therefore, patients frequently undergo both CT and MR imaging before surgery of cardiac masses. Moreover, future technical advances in T1 and T2 mapping sequences, radiomics, artificial intelligence and PET/MRI could allow for raising further MR accuracy to distinguish even better between non-neoplastic lesions, benign tumors and malignant neoplasms. 

However, in the future, spectral imaging could allow CT to become a comprehensive imaging modality without the need to perform a MR imaging for tissue characterization of cardiac lesions.

## Figures and Tables

**Table 1:** Cardiac pseudomasses.

Cardiac Lesion	Epidemiology	Location	Clinical Manifestations	Key Characteristics	Echocardiography	CT	MR
Coumadin ridge	Any age. Everybody	LA	Asymptomatic	Fold of the LA wall with a rounded edge between the left superior pulmonary vein and the left atrial appendage	nodular, pedunculated, or linear structure	Elongated structure similar to the atrial wall	Elongated structure similar to the atrial wall
Crista terminalis	Any age. Everybody	RA	Asymptomatic	Ridge in the posterolateral wall of the RA	Linear echogenic ridge	Linear ridge similar to the atrial wall	Linear ridge similar to the atrial wall
Taenia sagittalis	Any age. 80% of the population	RA, RAA	Asymptomatic	Ridge in the anterolateral wall of the RA	usually not visualized	Linear structure similar to the atrial wall	Linear structure similar to the atrial wall
Chiari network	Any age. 2–14% of the population	RA	Asymptomatic	lacelike structure in the RA over the IVC ostium or a linear band from the eustachian and/or thebesian valves to the crista terminalis	Reported in 2% of the population, mobile echogenic lacelike structure or a linear band	lacelike structure in the RA or a linear band	lacelike structure in the RA or a linear band
Moderator band	Any age, up to 92% of patients	RV	Asymptomatic	prominent muscular ridge in the RV	RV stripe	Muscular ridge similar to the RV wall	Muscular ridge similar to the RV wall
Papillary muscles	Any age. Everybody	LV, RV	Asymptomatic	from the inner wall of the ventricles in their middle or apical portions, with an elongated and tapered trunk	Echogenic elongated structures	Elongated structures similar to the LV and RV walls	Elongated structures similar to the LV and RV walls
LHIS	Late adulthood. Obesity	IAS	Usually, asymptomatic. Atrial arrhythmias	mass like deposition of brown fat in the IAS which spares the fossa ovalis	Homogeneous hyperechoic dumbbell appearance of atrial septum	mass with fat-attenuation which spares the fossa ovalis	Hyper T1w and T2w, no LGE, hypo on STIR and other fat-suppression sequences

Abbreviations–IAS: interatrial septum; LA: left atrium; RA: right atrium; RAA: right atrium appendage; RV: right ventricle; LHIS: lipomatous hypertrophy of the interatrial septum.

**Table 2:** Cardiac non-neoplastic lesions.

Cardiac Lesion	Epidemiology	Location	Clinical Manifestations	Key Characteristics	Echocardiography	CT	MR
Thrombus	Adulthood	LA, LAA (AF) LV (MI)	Asymptomatic, embolic events	Non-enhancing Intracardiac lesion	Acute: Low echodensity, rounded with smooth contours Chronic: High echodensity, linear or crescentic lesions along the endocardial surface	Low attenuation, no contrast enhancement, chronic thrombus may be calcified Usefulness of delayed CT imaging, as in the LAA stasis of blood can simulate a thrombus on early arterial images	Acute: hyper T1w and T2w Subacute: hyper T1w and hypo T2w Chronic: low T1w and T2w No enhancement.
Vegetations	Adulthood	Valves	Valve dysfunction, emboli, heart failure	Highly mobile, non-enhancing	Highly mobile, oscillating, protruding, valve dysfunction	Low attenuation, may recognize, perivalvular extension, fistulas, abscess	Highly mobile
Mitral annular calcification	Old patients	annular fibrous ring of the mitral valve	Asymptomatic	Calcifications, mitral valve	Hyperechoic	Calcific mass	Hypo T1w, hypo T2w, peripheral rim enhancement
Caseous degeneration of mitral annular calcification	Old patients	annular fibrous ring of the left atrio-ventricular valve	Asymptomatic	Calcifications, mitral valve	hyperechoic	Calcifications within and around the mass	Mildly hyper T1w, mildly hyper T2w, peripheral rim enhancement sometimes with central enhancement
Pericardial cyst	Adulthood	Right pericardiophrenic angle	Asymptomatic	Fluid-filled, thin-walled, homogeneous, no internal enhancement	Low echodensity	Hypodense well-defined lesion with a wall	Hypo T1w, hyper T2w, no internal enhancement
Coronary artery aneurisms	Adulthood	AV groove	Asymptomatic	Vascular mass	AV groove mass	Dilatation, thrombus, fistula	Vascular enhancement
Cardiac aneurysm and pseudoaneurysm	Adulthood	Cardiac chamber	heart failure	Blood-filled	Dilatation	Dilatation, thrombus	Dilatation, thrombus
IgG4-related disease	Adulthood Male to female ratio is 2:1.	AV groove and right atrial infiltration, pericardium, coronary arteries	pericardial tamponade, constrictive pericarditis, valvula stenosis or regurgitation	Coronary periaortitis, AV groove and right atrial infiltration, associated pancreatic, biliary, renal involvement	Pericardial effusion with highly echogenic thickening of the pericardium, valvular stenosis or regurgitation, pulmonary arterial hypertension	aortitis, periarteritis, coronary aneurysms, perivascular infiltration with coronary stenosis	Cardiac masses, pericardial thickening, valvular disease
Sarcoidosis	Adulthood	Basal septum, AV groove	AV block, LVEF reduction	Basal septal thickening, delayed myocardial enhancement, associated mediastinal/hilar adenopathy	Septal mass lesion	Basal septum thickening, rarely soft tissue infiltration into AV groove encasing the coronary artery	LGE, Active disease (oedema): hyper T2w
Foreign body	Adulthood	Intracardiac, pericardium	Usually asymptomatic; rarely life-threatening and highly symptomatic	Intracardiac or pericardial mass. Previous surgical or intravascular procedures	Hyperechoic, sometimes with posterior acoustic shadowing	High-density. Gossypiboma: heterogeneous, cystic	Gossypiboma: hypo T1w, hyper T2w with internal low signal intensity stripes Others: usually hypo T1w and T2w with possible surrounding artifacts
Hematoma	Usually adulthood, previous surgery, traumatic heart injury, coagulopaty, anticoagulants	Pericardium	Usually, asymptomatic	Previous cardiac surgery, mass near surgical site or clips, usually well-defined borders. Usually absent contrast-enhancement	Acute phase: echo-lucent Chronic phase: mass like and echogenic	Heterogeneous, clips, hyperdense in acute phase, density decrease in chronic phase, calcific components in chronic hematoma	Acute: hyper T1w and T2w Subacute: heterogeneous with hyper T1 and T2w areas Chronic: hypo T1w and T2w with dark peripheral rim. No internal enhancement, possible rim enhancement
Echinococcus cyst	Adulthood	Myocardium, pericardium	Usually, asymptomatic	Typical hydatic cyst imaging characteristics	Well defined cystic mass with or without septations	Hypodense lesion with a wall, daughter cysts, peripheral calcifications	Hypo T1w, hyper T2w

Abbreviations–AF: atrial fibrillation; AV: atrioventricular; LA: left atrium; LAA: left atrium appendage; LVEF: left ventricular ejection fraction; MI: myocardial infarction; hyper: hyperintense; hypo: hypointense.

**Table 3:** Cardiac benign tumors.

Cardiac Lesion	Epidemiology	Location	Clinical Manifestations	Key Characteristics	Echocardiography	CT	MR
Myxoma	Adulthood. Carney complex.	LA	Usually, asymptomatic. Rarely, intracardiac obstruction, embolic events and constitutional symptoms	Mobile mass arising from the IAS	Globular or spherical, with a friable surface and heterogeneous internal echogenicity	Heterogeneous, low attenuation, may be calcified	Isointense T1w, High T2w, heterogeneous LGE
Papillary fibroelastoma	Adulthood	Valves	Usually, asymptomatic. Rarely embolic events	Atrial side of the mitral valve or the aortic surface of the aortic valve leaflet	Stippling and vibration or shimmer of the peripheral edge.	Hypodense, smooth, peduncolated, attached to the valve leaflet by a short pedicle	Iso T1w, Hyper T2w, hypo cine with surrounding turbolent flow, poor LGE
Lipoma	Adulthood Tuberous sclerosis	LV, any other site	Asymptomatic. If large, sometimes arrhythmias or obstructive symptoms	Circumscribed homogeneous fat-containing mass	Homogeneous, hyperechoic in the cavity but hypoechoic in the pericardium	Smooth, homogeneous mass with fat-attenuation	Hyper T1w and T2w, no LGE, hypo on STIR and other fat-suppression sequences
Rhabdomyoma	Fetal life and childhood. Tuberous sclerosis	LV, IVS	Asymptomatic. Rarely flow obstruction, heart failure, arrhythmias	Intramyocardial masses, frequently multiple	Homogenous, slightly echogenic	Attenuation similar to myocardium	Iso T1w, iso-hyper T2w, no or minimal enhancement
Fibroma	Childhood. Gorlin Sd	LV, IVS	Asymptomatic. Rarely arrhythmias	Intramyocardial mass, solitary	Heterogeneous, echogenic, non-contractible, can mimic HCM	Soft tissue attenuation, low contrast enhancement, may show central calcification	Iso-hypo T1w, hypo T2w, no or minimal enhancement in perfusion imaging. High LGE
Hemangioma	Adulthood	RA, RV, LV, any other site including pericardium	Usually, asymptomatic.	Inhomogeneous. Evident post-contrast enhancement. Well-demarcated without invasion of adjacent tissues and structures	hyperechogenic mass	water attenuation. Rarely, calcifications	Iso T1w, hyper T2w, strong enhancement in perfusion imaging, high LGE
Lymphangioma	Any age	Pericardium, any other site	respiratory distress/dyspnea, arrhythmia, chest pain, heart failure	Mass with cystic and septal components	Echogenic mass with cystic and septal components	Cysts, absence of calcifications or macroscopic fat.	Hypo T1w, hyper T2w, enhancement of cystic walls and septa, no internal enhancement
Erdheim-Chester disease	Adulthood Male to female ratio is 3:1.	RA, AV groove, pericardium	Heart failure, myocardial infarction, tamponade	symmetric osteosclerosis of the metadiaphysis of the lower-extremity bones, RA masses, AV groove infiltration, periarterial infiltration	Pericardial effusion	Right atrium or AV sulcus mass, coronary periaortitis, pericardial thickening and effusion	Strong enhancement
Solitary fibrous tumor	Adulthood	Pericardium	Asymptomatic, dyspnea, palpitation	Slow-growing pericardial mass	inhomogeneous pericardial mass, pericardial effusion	Low attenuation, scarce enhancement	Iso T1w, heterogeneous or homogeneous hyper T2w, hyper on SPIR, septated or patchy LGE
Teratoma	Fetal life and childhood.	pericardium	compression on the heart and cardiac tamponade	Mass with cysts, gross fat, calcification, and regions of soft tissue.	Heterogeneous	well-defined mass with a variable extent of cystic degeneration, calcification, and areas of fatty tissue	gross fat: hyper T1w and T2w, with signal decrease on a fat-suppressed T2w
Schwannoma	Adulthood. Schwannomatosis	RA, right atrioventricular groove	Usually, asymptomatic. Rarely, chest tightness, dyspnea, cough, fatigue, and facial edema	circumscribed, slow enhancement	Heterogeneous, echogenic mass	Low attenuation, scarce enhancement, scattered calcifications	Iso-hypo T1w, target appearance on T2w images with central hypointensity and peripheral hyperintensity. Slow internal enhancement
Paraganglioma	Adulthood	On the roof of left atrium, right atrioventricular groove	elevated blood pressure and severe headaches	“salt and pepper” appearance, highly vascular, parasitizes blood supply from coronary arteries, elevated serum metanephrines Malignant up to 25%	Solid heterogeneous echogenic mass with clear boundaries, and detectable blood flow signals inside it in the color Doppler	A mass of soft-tissue density with homogeneous (smaller lesions) or peripheral (larger lesions with hemorrhage, necrosis and cystic degeneration) enhancement	Iso-hypo T1w, hyper T2w, peripheral LGE. Perfusion imaging shows strong enhancement

Abbreviations–AV: atrioventricular; IAS: interatrial septum; IVS: interventricular septum; LA: left atrium; LV: left ventricle; RA: right atrium; RV: right ventricle; hyper: hyperintense; hypo: hypointense; iso: isointense; Sd: syndrome.

**Table 4:** Cardiac malignant tumors.

Cardiac Lesion	Epidemiology	Location	Clinical Manifestations	Key Characteristics	Echocardiography	CT	MR
Metastasis	Adulthood Melanoma, lung, breast and esophageal cancers common. Transvenous: HCC and RCC	Myocardium, pericardium, right atrium in transvenous spread	Flow obstruction/heart failure, arrhythmia, pericardial effusion	Multiple myocardial or pericardial masses. Right atrium lesions in transvenous spread. More often circumscribed lesions	Heterogeneous, highly echogenic with contrast infusion	Similar to soft tissue attenuation	Hypo-iso T1w, Iso-hyper T2w, heterogeneous LGE. Melanoma: hyper T1w
Angiosarcoma	Early and middle adulthood, Li-Fraumeni Sd	RA near AV sulcus, pericardium	Constitutional symptoms, heart failure, pericardial effusion	Vascular nature; hemorrhage and necrosis. Lung, liver and brain metastasis.	Iso-hyperechogenic and irregular mass, often as a nonmobile, broad-based, endocardial neoplasm with myocardial extension. Pericardial effusion	low-attenuation, irregular, intracavitary mass is often shown, pericardial thickening with effusion, heterogeneous enhancement	Heterogeneous Iso-hyper T1w, heterogeneous-hyper T2w, marked and heterogeneous enhancement and LGE. Perfusion imaging in the arterial phase shows immediate and strong enhancement
Undifferentiated pleomorphic sarcoma (UPS)	Early and middle adulthood, Li-Fraumeni Sd	LA	Flow obstruction/heart failure, pericardial effusion, metastatic	Endocardial growths that protrude into the chamber and invade the adjacent myocardium. Distant metastasis	Heterogeneous, normally-highly echogenic	Heterogeneous, large, irregular low attenuation	Heterogeneous iso T1w, hyper T2w and LGE. Heterogeneous enhancement at first pass
Leiomyosarcoma	Adulthood, Li-Fraumeni Sd	LA	Flow obstruction/heart failure, pericardial effusion	Distant metastasis and local recurrence	Echogenic and irregular mass	irregular low attenuation	Iso-hypo T1w, hyper T2w, heterogeneous LGE. Heterogeneous enhancement at first pass
Rhabdomyosarcoma	Childhood, early adulthood	LV, RV	Heart failure, arrhythmia, eosinophilia	Multiple myocardial masses	Normally-highly Echogenic	Irregular, low attenuation	Iso T1w, T2w, hyper STIR, homogeneous LGE. Enhancement at first pass
Lymphoma	Adulthood, Immunocompromised	RA, RV, pericardium	Pericardial effusion, flow obstruction/heart failure	Uniform, infiltrative, extend along the epicardial surfaces of the heart, encase adjacent vascular structures, hypermetabolic on PET	Pericardial effusion. Tumor might be detected with homogeneous echogenicity.	Pericardial effusion. Normal to low attenuation, heterogeneous and mild contrast enhancement	Iso-hypo T1w, Iso-hyper T2w, none-minimal homogeneous LGE. Evident diffusion restriction
Mesothelioma	Adulthood	Pericardium	constrictive pericarditis, tamponade	Diffuse pericardial involvement with complete encasement of the heart	Pleural effusion with echogenic lesions	Diffuse irregular pericardial thickening with heterogeneous enhancement	Iso T1w, heterogeneous T2w, heterogeneous LGE

Abbreviations–AV: atrioventricular; LA: left atrium; LV: left ventricle; RA: right atrium; RV: right ventricle; RCC: renal cell carcinoma; HCC: hepatocellular carcinoma; hyper: hyperintense; hypo: hypointense; iso: isointense; Sd, syndrome.

## Data Availability

Data is contained within the article.
